# Optimizing the evaluation of gene-targeted panels for tumor mutational burden estimation

**DOI:** 10.1038/s41598-021-00626-7

**Published:** 2021-10-26

**Authors:** Yawei Li, Yuan Luo

**Affiliations:** Department of Preventive Medicine, Feinberg School of Medicine, Northwestern University, Chicago, IL 60611 USA

**Keywords:** Cancer, Tumour immunology

## Abstract

Though whole exome sequencing (WES) is the gold-standard for measuring tumor mutational burden (TMB), the development of gene-targeted panels enables cost-effective TMB estimation. With the growing number of panels in clinical trials, developing a statistical method to effectively evaluate and compare the performance of different panels is necessary. The mainstream method uses R-squared value to measure the correlation between the panel-based TMB and WES-based TMB. However, the performance of a panel is usually overestimated via R-squared value based on the long-tailed TMB distribution of the dataset. Herein, we propose angular distance, a measurement used to compute the extent of the estimated bias. Our extensive in silico analysis indicates that the R-squared value reaches a plateau after the panel size reaches 0.5 Mb, which does not adequately characterize the performance of the panels. In contrast, the angular distance is still sensitive to the changes in panel sizes when the panel size reaches 6 Mb. In particular, R-squared values between the hypermutation-included dataset and the non-hypermutation dataset differ widely across many cancer types, whereas the angular distances are highly consistent. Therefore, the angular distance is more objective and logical than R-squared value for evaluating the accuracy of TMB estimation for gene-targeted panels.

## Introduction

Immunotherapy has become an increasingly available and important cancer treatment option in recent years. Unlike chemotherapy that acts directly on cancer cells, immunotherapy uses a person's own immune system to fight cancer through a variety of forms, including targeted antibodies, cancer vaccines, adoptive cell transfer, tumor-infecting viruses, checkpoint inhibitors, cytokines, or adjuvants. However, immunotherapy is not yet as widely used as surgery, chemotherapy or radiation therapy, as it is likely to benefit some people with certain types of cancers like melanoma, lung and kidney cancer^1^. To determine whether immunotherapy is effective, studies have incorporated (to varying degrees) several biomarkers into clinical practice, including PD-1/PD-L1 expression^2^, CTLA-4 expression^3^, CD8^4^, CD39^5^, mismatch repair (MMR) and microsatellite instability (MSI)^6,7^. Similar to the previous biomarkers, the tumor mutational burden (TMB) is a predictive biomarker with the potential to make a significant impact on cancer immunotherapy.

TMB, a measurement of the mutations carried by tumor cells, has been widely used to evaluate their association with response to Immuno-Oncology (I-O) therapy^8,9^, including the associations between different TMB levels and patient response to immune checkpoint blockade (ICB) therapy in a variety of cancers^10–12^. In 2014, Snyder et al.^13^ first analyzed the correlation between TMB and treatment effectiveness by analyzing whole-exon sequencing (WES) data from melanoma patients treated with anti-CTLA-4. Later, Rizvi et al.^14^ used therapies with anti–PD-1 in non–small cell lung cancer (NSCLC) patients and demonstrated that higher nonsynonymous mutational burden in tumors was associated with improved objective response, durable clinical benefit, and progression-free survival (PFS). More recently, studies found that combining TMB with these published cell surface checkpoint inhibitors (PD-1, PD-L1, CTLA-4) improves the accuracy of ICB therapy outcome prediction. For example, NSCLC patients with PD-L1 ≥ 5% and a high tumor mutational burden seemed to benefit from nivolumab administration that had a longer median PFS^15^. The success of using TMB to predict ICB therapy is that higher TMB values usually result in a higher number of neoantigens presenting on the tumor cell surface^10^. As a result, these higher numbers of neoantigens increase the chance of being detected from ICB therapy^10^. However, a recent study found that using TMB to predict ICB response may not be suitable for all cancer types^16^. Additional investigation is needed to improve the utility of TMB for accurate prediction of response to ICB. Apart from immunotherapy, TMB was also reported to be associated with other cancer therapies both in clinically^17,18^ and theoretically^19,20^.

Typically, WES allows comprehensive TMB estimation, and is considered the “gold-standard”. However, it is impossible for all patients to use WES to estimate their TMBs due to the high cost of the large genomic space sequenced. To this end, the development of gene-targeted panels enables cost-effective TMB estimation for more patients. Clinical trials have shown that TMB can be estimated from targeted sequencing of only a few hundred genes of interest, which can be performed at a high depth for clinical applications. The next generation sequencing (NGS) panel FoundationOne CDx (F1CDx) developed by Foundation Medicine, counting both synonymous mutations and nonsynonymous mutations (not included in dbSNP or COSMIC database) from 324 genes (1.2 Mb), has proven highly consistent with the estimation from WES^21–23^. Another panel MSK-IMPACT, targeting only nonsynonymous mutations from 468 genes (1.22 Mb), also shows a high correlation with the results from WES^24^. Based on their valuable performance, they have been approved by the FDA as pan-cancer genomic profiling tests. In the meantime, more commercially available genomic profiles are being developed for TMB estimation.

With the growing number of gene-targeted panels, developing a statistical method to effectively evaluate and compare the performance of different panels is necessary. As an important criterion, the estimated TMB is expected to be close to WES-based TMB. The mainstream statistical method uses the coefficient of determination (R-squared) to evaluate a panel. R-squared value is a statistical measure that explains the strength of the correlation between an independent and dependent variable via a regression model. However, our comprehensive analysis demonstrates that the R-squared value often overestimates the performance of a panel due to the unbalanced TMB distribution of patients. Herein, we used angular distance as opposed to R-squared, which performs a more veritable, objective and logical measurement when evaluating the performance of a panel.

## Results

### Using angular distance rather than linear regression to evaluate gene-targeted panels

R-squared is the percentage of the response variable variation that is explained by a linear model, which measures how close the data are to the fitted regression line. Under the definition of R-squared, the estimated bias of the panel cannot be directly measured by R-squared. For example, let $x_{1}$, $x_{2}$, …, $x_{n}$, denote the TMBs estimated by a gene-targeted panel and $y_{1}$, $y_{2}$, …, $y_{n}$, denote the WES-based TMBs of the dataset. The linear regression function is,

1 $$\begin{array}{*{20}c} {y = ax + b} \\ \end{array}$$

where *a* and *b* can be solved by,

2 $$\begin{array}{*{20}c} {\mathop {\arg \min }\limits_{a, b} \frac{1}{2}\mathop \sum \limits_{i = 1}^{n} \left( {y_{i} - ax_{i} - b} \right)^{2} } \\ \end{array}$$

By solving the partial derivatives, *a* and *b* yield,

3 $$\begin{array}{*{20}c} {\left\{ {\begin{array}{*{20}l} {a = \frac{{\mathop \sum \nolimits_{i = 1}^{n} \left( {x_{i} - \overline{x}} \right)\left( {y_{i} - \overline{y}} \right)}}{{\mathop \sum \nolimits_{i = 1}^{n} \left( {x_{i} - \overline{x}} \right)^{2} }} = \frac{{\mathop \sum \nolimits_{i = 1}^{n} x_{i} y_{i} - n\overline{x}\overline{y}}}{{\mathop \sum \nolimits_{i = 1}^{n} x_{i}^{2} - n\overline{x}^{2} }}} \hfill \\ {b = \overline{y} - a\overline{x}} \hfill \\ \end{array} } \right.} \\ \end{array}$$

where $\overline{x}$ and $\overline{y}$ denote the average value of $x_{1}$, $x_{2}$, …, $x_{n}$ and $y_{1}$, $y_{2}$, …, $y_{n}$. , respectively. According to Eq. (1), *a* is the slope of the linear function and *b* is the Y-intercept number. The R-squared value can be represented as,

4 $$\begin{array}{*{20}c} {R^{2} = 1 - \frac{{\mathop \sum \nolimits_{i = 1}^{n} \left( {y_{i} - ax_{i} - b} \right)^{2} }}{{\mathop \sum \nolimits_{i = 1}^{n} \left( {y_{i} - \overline{y}} \right)^{2} }}} \\ \end{array}$$

**Figure 1:**
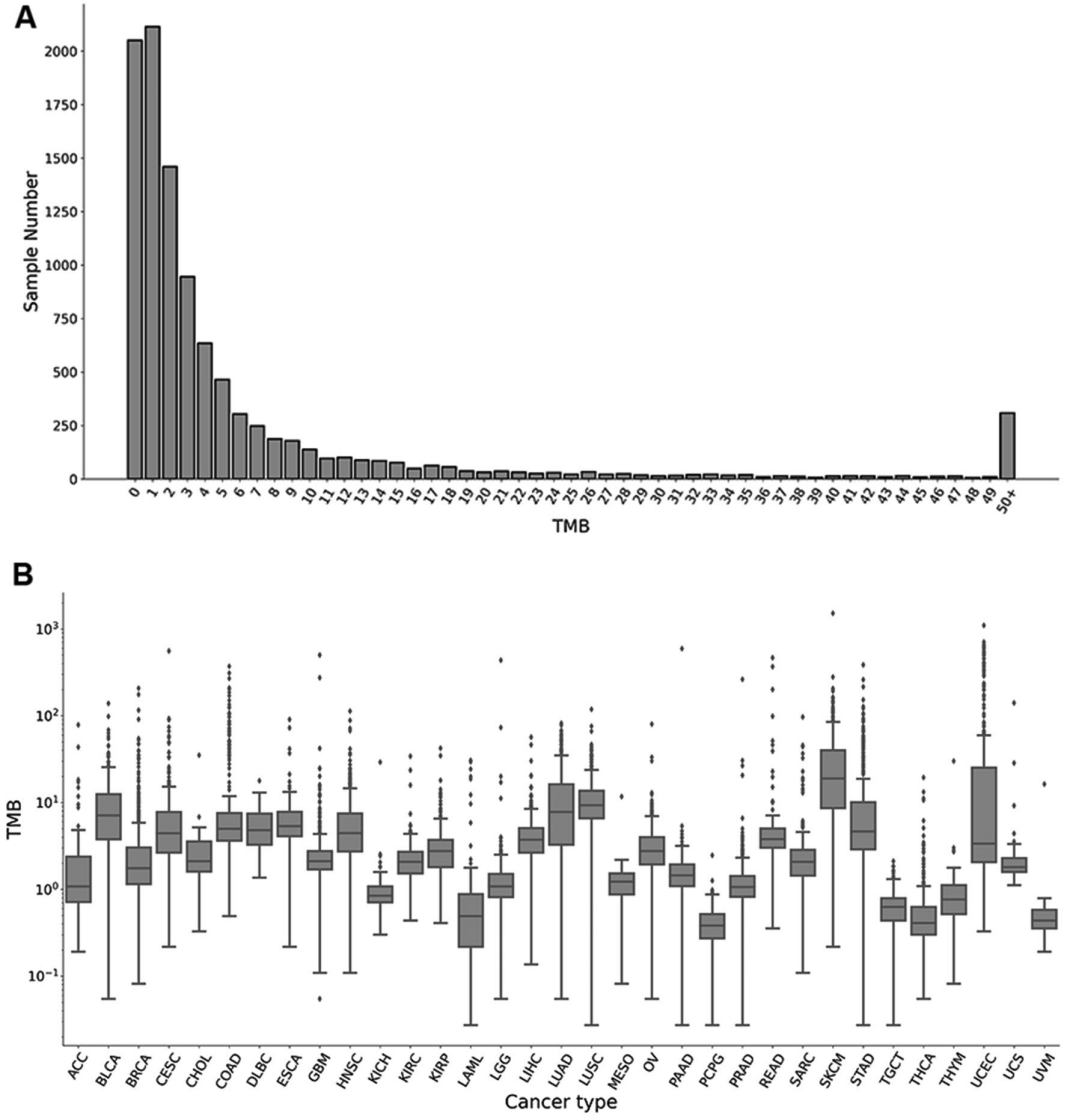
The TMB distribution of the 10,223 patients across 33 different cancer types. (**A**) The histogram of the TMB distribution of all patients. The X axis is the TMB (the number of somatic mutations per megabase (Mb) of interrogated genomic sequence), and the Y axis is the number of patients with the integer TMB being the corresponding number. The average TMB for the dataset is 9.64, and the median TMB is 2.60. (**B**) The boxplot of the TMB distribution for each of the 33 cancer types. The X axis denotes the 33 cancer types and the Y axis is the TMB for each cancer type. A log-10 scale is used for the Y axis of the graph.

The denominator of Eq. (4) is the total sum of squares, which is influenced by the TMB distributions of the entire dataset. Nevertheless, in terms of the Cancer Genome Atlas (TCGA) dataset, the TMB distribution presents a long-tailed pattern across all cancer types (Fig. 1). The average TMB value for the entire dataset is 9.64 mutations/Mb, but approximately 83% of the patients are below the average value. In contrast, the average value of the hypermutated patients (TMB > 50) reaches 151.51. Due to these hypermutated patients, the variance of the total dataset reaches 1559.63, which leads to an extremely high denominator in Eq. (4). The numerator of the Eq. (4) represents the sum of the squared residuals. Ideally, we expect that $x_{i}$ should be very close to $y_{i}$ for each patient *i*. If the Y-intercept (*b*) in the numerator is not close to 0, for a patient with panel-based TMB $x_{m}$ and WES-based TMB $y_{m}$, its estimated bias is $y_{m} /x_{m} \approx a + {\text{b}}/x_{m}$. The bias will be very large if TMB of the patient ($x_{m}$) is very small. According to the TMB distribution of the TCGA dataset, a total of 20.03% of the patients are within interval [0, 1), and another 20.66% of patients are within interval [1, 2) (Fig. 1A). As a result, the high bias rate of these lower-TMB patients indicates that the performance of the panel is poor. If the Y-intercept (*b*) is ≈0, then considering two patients, their WES-based TMBs are $y_{m}$ and ${\text{k}}y_{m}$ (k >> 1), and their panel-based TMBs are $x_{m}$ and ${\text{k}}x_{m}$, respectively. The ratios of the panel-based TMB to WES-based TMB for the two patients are both equal to $y_{m} /x_{m}$. However, the squared residual of the higher-TMB patient ($\approx k^{2} \left( {y_{m} - ax_{m} } \right)^{2}$) is about $k^{2}$-folds higher than the lower-TMB patient ($\approx \left( {y_{m} - ax_{m} } \right)^{2}$), indicating that the contribution of the higher-TMB patient is $k^{2}$-folds higher than the lower-TMB patient in the R-squared value. As a consequence, R-squared value is mainly determined by those extremely high TMB patients in the dataset.

In light of the limitations of R-squared measurement, we developed a new metric, the angular distance, to measure the estimated bias for a panel. For a patient *i*, denote $x_{i}$ and $y_{i}$ are the panel-based TMB and WES-based TMB, respectively. So, the Cartesian coordinate of the patient is ($x_{i} ,y_{i}$). If we transfer the Cartesian coordinate to the polar coordinates, the coordinate of the patient ($r_{i} ,\varphi_{i}$) can be derived as follows,

5 $$\begin{array}{*{20}c} {\left\{ {\begin{array}{*{20}l} {r_{i} = \sqrt {x_{i}^{2} + y_{i}^{2} } } \hfill \\ {\varphi_{i} = \arctan \left( {\frac{{y_{i} }}{{x_{i} }}} \right)} \hfill \\ \end{array} } \right.} \\ \end{array}$$

In terms of Eq. (5), if the predicted panel-based TMB (${\text{x}}$) is absolutely equal to the WES-based TMB (${\text{y}}$), the expected angle ($\varphi$) is equal to $\pi /4$. Therefore, the angular distance (estimated bias, ${\uptheta }_{i}$) of patient *i* can be expressed as:

6 $$\begin{array}{*{20}c} {\uptheta _{i} = \left| {\frac{\pi }{4} - \varphi_{i} } \right| = \left| {\frac{\pi }{4} - \arctan \left( {\frac{{y_{i} }}{{x_{i} }}} \right)} \right|} \\ \end{array}$$

Theoretically, the angular distance $\uptheta _{i}$ ranges from 0 (the estimated panel-based TMB by is equal to the WES-based TMB) to $\pi /4$ (the estimated TMB by gene-targeted panel is equal to 0) based on Eq. (6). Lower angular distance represents a smaller estimated bias. We use the average value of $\uptheta _{i}$ ($\uptheta = \sum\uptheta _{i} /n$) to measure the overall performance of a gene-targeted panel. Compared with the R-squared value that computes the Euclidean distance of each patient, and is primarily determined by the patients with extremely high TMB, the benefit of angular distance is that the weight of each patient contributing to measuring the performance of a panel is absolutely equal.

### Renowned commercially gene-targeted panel evaluation by the two measurements

**Figure 2:**
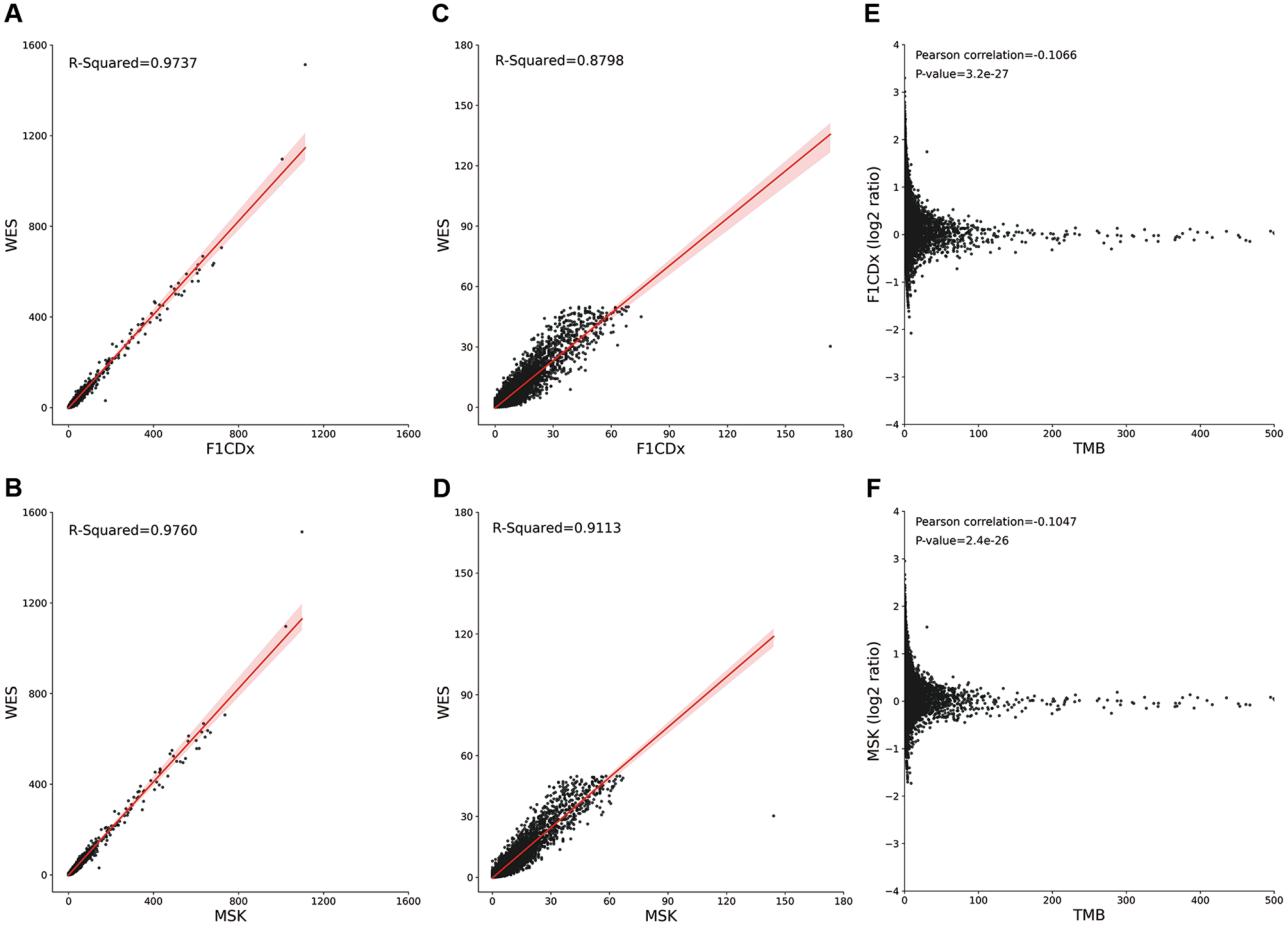
TMB estimation results for gene panels in different datasets. (**A**, **B**) Linear fit with 95% confidence intervals of panel-based TMB estimated by the F1CDx and the MSK against WES-based TMB in the hypermutation-included dataset. (**C**, **D**) Linear fit with 95% confidence intervals of panel-based TMB estimated by the F1CDx and the MSK against WES-based TMB in the non-hypermutation dataset. The R-squared values are lower in the non-hypermutation dataset than in the hypermutation-included dataset for both panels. (**E**, **F**) The correlation between the estimated bias and TMB size (Pearson correlation: F1CDx: − 0.1066, *P *value: 3.2 × 10^–27^; MSK: − 0.1047, *P *value: 2.4 × 10^–26^). The X axis is the WES-based TMB of the patient and the Y axis is calculated as the panel-based TMB divided by the WES-based TMB. A log-2 scale is used for the Y axis of the graph. Patients with TMB > 500 are not shown.

We first employed R-squared and angular distance to compare and evaluate seven (see Methods) renowned commercially gene-targeted panels. The rankings of the performance for the seven panels (OTML > TSO500 > QIAseq > MSK > F1CDx > OCAv3 > TST170) were absolutely the same between the two measurements (Table 1). However, in the hypermutation-included datasets, the R-squared values ranged from 0.9598 to 0.9842 (Fig. 2A, B and Fig. S1), whereas the R-squared values were much lower (0.8176 ~ 0.9298) when we omitted the hypermutated patients (Fig. 2C, D and Fig. S2). Lower R-squared values in the non-hypermutation dataset than in the hypermutation-included dataset suggested that the estimation was more accurate in higher TMB patients. We calculated the estimated bias by calculating the panel-based TMB divided by the WES-based TMB for each patient (Fig. 2E, F and Fig. S3). The estimated biases for many lower-TMB patients were very high, whereas the biases for higher-TMB patients were much lower. Though the proportion of these higher-TMB patients was low, the R-squared value was still high since the squared residuals of these higher-TMB patients were large in Eq. (4). In contrast to the R-squared, the angular distances between the hypermutation-included dataset and the non-hypermutation dataset were similar across all seven panels (Table 1), indicating that angular distance was more robust than R-squared as a measurement.

**Table 1:** The R-squared values and average angular distances for the selected panels.

Panel	Hypermutation-included dataset		Non-hypermutation dataset	
	R-squared	Angular distance	R-squared	Angular distance
F1CDx	0.9737	0.3106	0.8798	0.3182
MSK-IMPACT	0.9760	0.2705	0.9113	0.2769
OCAv3	0.9610	0.3809	0.8262	0.3901
OTML	0.9842	0.2483	0.9298	0.2546
TSO500	0.9810	0.2585	0.9272	0.2648
TST170	0.9598	0.3872	0.8176	0.3966
QIAseq	0.9776	0.2629	0.9214	0.2694

Previous studies have demonstrated that TMB was distributed widely across different cancer types^22,25,26^. To this end, we used the two measurements to evaluate the performance of the panels in each of the 33 cancer type datasets (Table S1, S2, S4 and S5, Fig. S4 and S5). The R-squared values varied greatly between different cancer types for all seven panels (Table S1). In the hypermutation-included dataset, three cancer types, READ, PAAD and UCEC, achieved average R-squared values > 99%. In contrast, three other cancer types, OV, PCPG and TGCT performed poorly, with R-squared values < 70% in most selected panels. Comparing the R-squared values between the two datasets across cancer types, the R-squared values were lower in the non-hypermutation dataset for most cancer types (Table S2). Strikingly, four cancer types, GBM (Fig. S6), LGG (Fig. S7), PAAD (Fig. S8) and PRAD (Fig. S9), achieved very high average R-squared values (0.9880, 0.9696, 0.9944 and 0.9787; Table S1) in the hypermutation-included datasets, but much lower values in the non-hypermutation datasets (0.8164, 0.6942, 0.8096 and 0.7860; Table S2). In fact, among the 392 GBM patients, 512 LGG patients, 177 PAAD patients and 497 PRAD patients, only two GBM patients, two LGG patients, one PAAD patient and one PRAD patient were hypermutated. In all four cancer types, the TMB of the hypermutated patients exceeded 250 mutations/Mb, which was more than 100-fold higher than the average TMB of the remaining patients. Omitting the hypermutated patients, the variances of the dataset for the four cancer types were 11.89 (GBM), 1.78 (LGG), 0.81 (PAAD) and 4.31 (PRAD), respectively, whereas the variances became 828.97 (GMB), 379.51 (LGG), 1978.26 (PAAD) and 142.10 (PRAD) when containing the hypermutated patients (Table S3). Based on Eq. (4), the increased total sum of squares led to a higher R-squared value in the hypermutation-included dataset.

**Figure 3:**
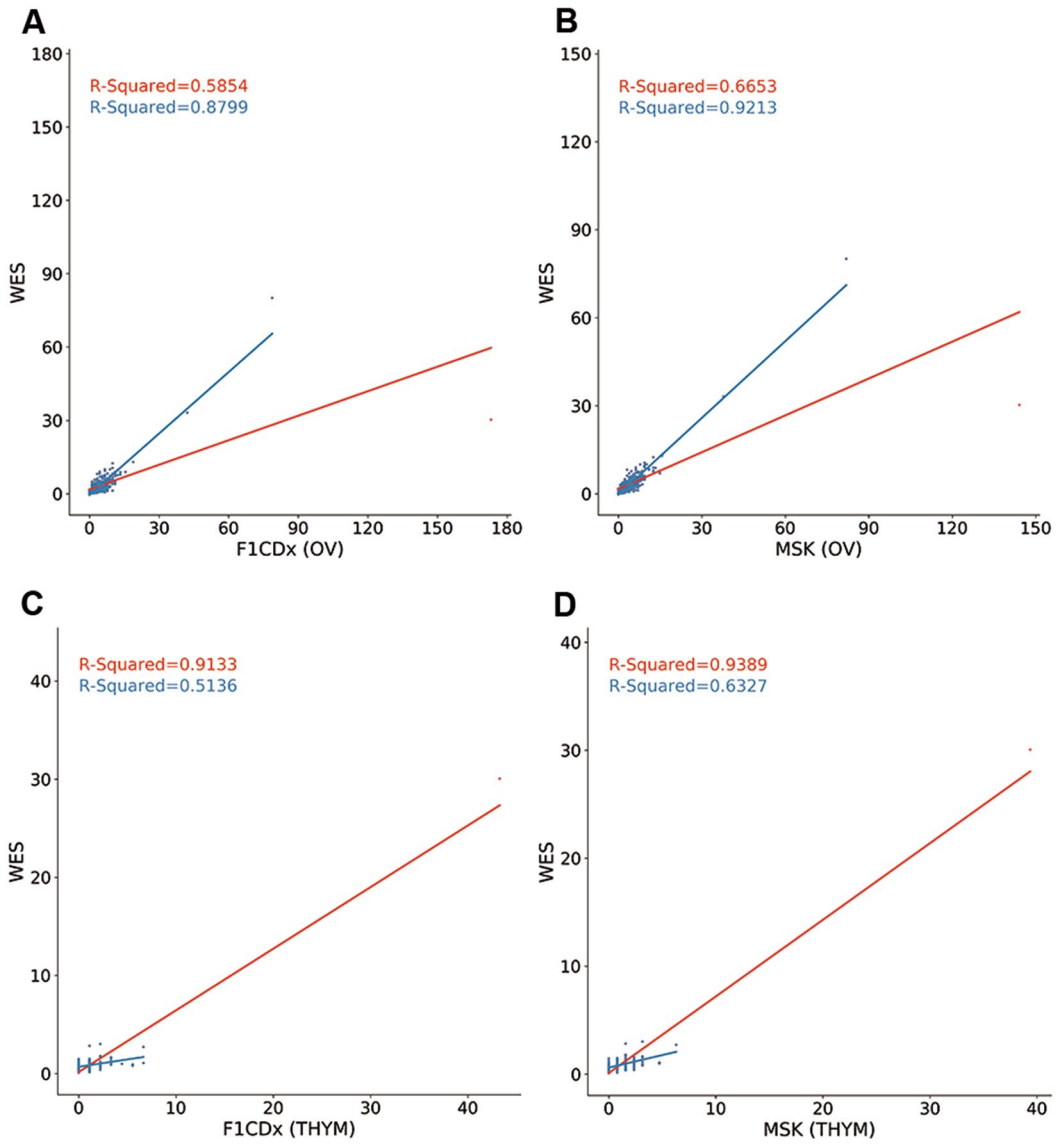
Linear fit of panel-based TMB estimated by the F1CDx (**A**, **C**) and MSK (**B**, **D**) against WES-based TMB in cancer type OV dataset (**A**, **B**) and THYM dataset (**C**, **D**). The poor R-squared value in the cancer type OV dataset is due to the highly estimated bias of one patient (TCGA-13-0889, red dot in **A**, **B**) with very high TMB relative to other patients in the dataset, whereas the high R-squared value in cancer type THYM dataset is because the estimated bias of the patient (TCGA-ZB-A966, red dot in **C**, **D**) with very high TMB relative to other patients in the dataset is small.

The average angular distances of the panels ranging from 0.1314 to 0.5918 in the hypermutation-included dataset (Table S4) and from 0.1424 to 0.5918 in the non-hypermutation dataset (Table S5) were similar across the 33 cancer types. In particular, unlike R-squared, the average angular distances of GBM, LGG, PAAD and PRAD in the hypermutation-included dataset (0.2984, 0.4674, 0.4264 and 0.4216) were nearly the same as in the non-hypermutation dataset (0.2996, 0.4674, 0.4264 and 0.4216). Strikingly, we found that the two measurements were completely opposite in two cancer types: OV and THYM. The average angular distance in OV dataset was low, but the average R-squared value indicated that the estimation was poor. In contrast, the average angular distance in THYM dataset was very high, but the average R-squared value indicated that the estimation was good. To resolve the apparent contradiction, we analyzed the linear fit of the dataset for the two cancer types (Fig. 3). The low R-squared value in OV dataset was due to the highly estimated bias of one patient (TCGA-13-0889, red dot in Fig. 3A, B). After we omitted this patient, the average R-squared value increased to 0.8799. For cancer type THYM, its TMB distribution was similar to the four cancer types GBM, LGG, PAAD and PRAD: except for the highest TMB patient (TCGA-ZB-A966, red dot in Fig. 3C, D), the TMB of the second highest patient was only 3.01, about 10-folds lower than the highest one. Indeed, when we omitted this patient, the average R-squared value of the dataset was only 0.5571. Hence, angular distance was generally more accurate and objective than the R-squared value.

### The correlation between panel size and the performance

**Figure 4:**
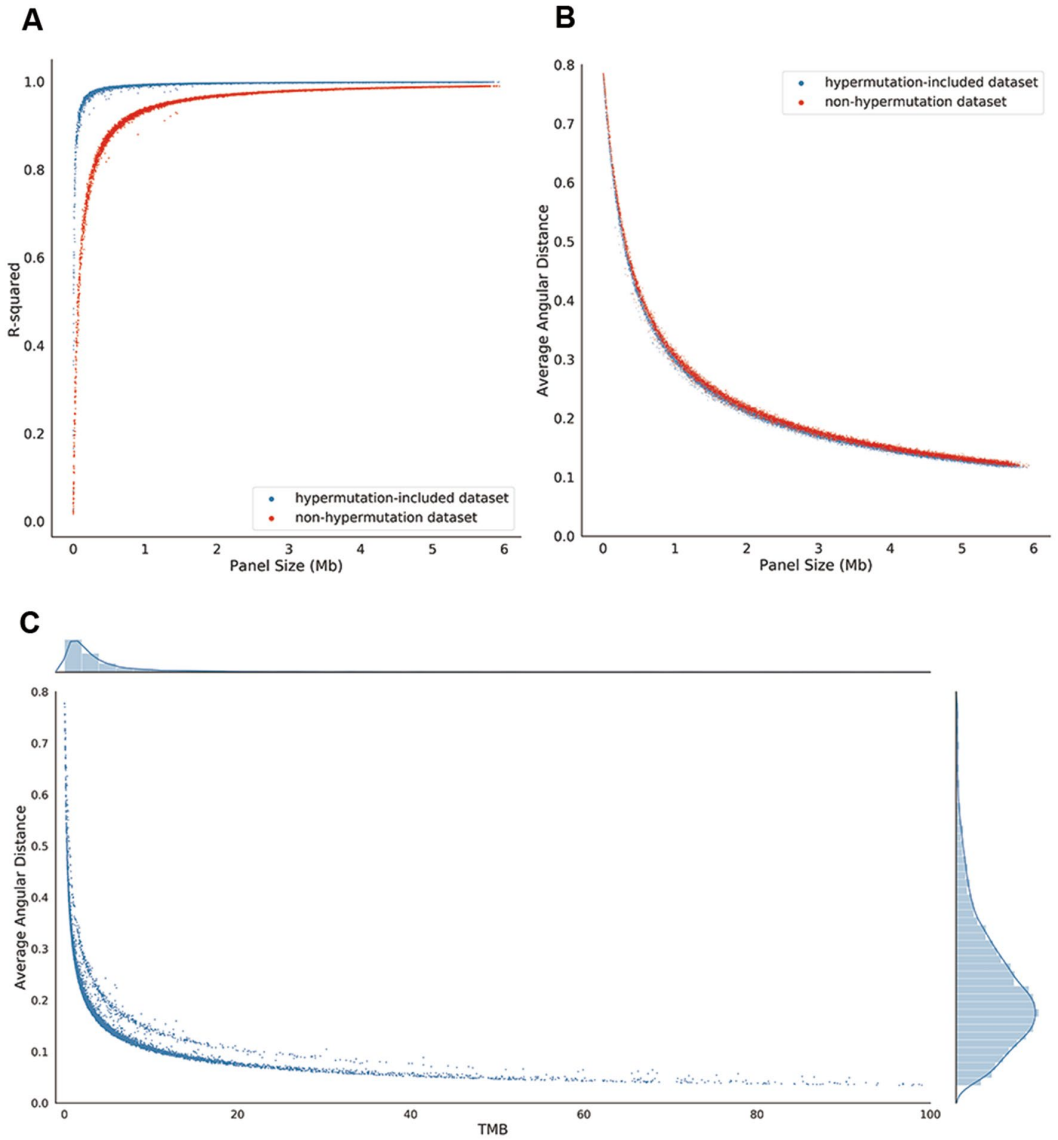
TMB estimation results for the 10,000 simulated sequencing panels in different datasets. (**A**) The R-squared of the simulated sequencing panels of the hypermutation-included dataset (blue) and the non-hypermutation dataset (red). The X axis is the size of each simulated panel, and the Y axis is the R-squared value of each panel. (**B**) The average angular distance of the simulated sequencing panels of the hypermutation-included dataset (blue) and the non-hypermutation dataset (red). The X axis is the size for each simulated panel, and the Y axis is the average angular distance estimated for each simulated panel. (**C**) The correlation between TMB and average angular distance. The X axis is the WES-based TMB of the patient and the Y axis is the average angular distance of the 10,000 simulated panels. Patients with TMB > 100 are not shown.

The rankings of the performance of the seven panels suggested that the performance of a panel may be associated with its panel size. To test our hypothesis, we randomly generated 10,000 panels in silico (see Methods) and analyzed the correlation of the two factors. Figure 4A, B display the correlations between the values of the two measurements (R-squared and angular distance) and panel size for the 10,000 simulated panels. With the increase in panel size, R-squared increases and angular distance decreases, indicating that panel size is an important factor to improve the accurate of TMB estimation, which is consistent with previous studies^27,28^. Comparing the changes in R-squared to the changes in angular distance with the increase in panel size, R-squared increases quickly and reaches a plateau after the panel size reaches 0.5 Mb. However, the angular distance of the panels shows that the estimated bias is still high when panel size is 0.5 Mb. Indeed, the angular distance is still sensitive to the changes in panel sizes when the panel size reaches 6 Mb. In addition, for most panels, their R-squared values between the hypermutation-included dataset and the non-hypermutation dataset are extensively different (Fig. 4A), but their angular distances between the two datasets are close (Fig. 4B). Moreover, by comparing the average angular distance of the 10,000 simulated panels to the real TMB of each patient, we find that their average angular distance is negatively correlated with their TMB (Fig. 4C). Therefore, for patients with higher TMBs, a smaller panel size sufficiently conforms to the estimation, while for patients with lower TMBs, a larger panel size is needed.

One of the major applications of TMB is that it is a biomarker for predicting ICB outcomes. In the study of CM026 trail, PFS and objective response rate (ORR) were significantly higher with nivolumab than with chemotherapy when the missense mutation count of the patient was no less than 243^15^. A similar study demonstrated that the PFS of patients whose TMB ≥ 10 mutations/Mb was significantly longer when using immunotherapy than chemotherapy^29^. Following the recent FDA approval of a 10 mutations/Mb threshold to select patients for ICB therapy, accurately estimating the patients with TMB ≈10 mutations/Mb is extremely important. To this end, we extracted the patients with TMB between 9 and 11 mutations/Mb, and scrutinized the correlations between their average angular distance and the panel size (Fig. S10) based on the observations of the 10,000 simulated panels. If the expected bias of the panels-based TMB to WES-based TMB was less than 20%, then the angular distance should be < 0.1107 (Eq. (6)), and the panel size needed to be > 1.30 Mb. If the expected bias was less than 10% (angular distance < 0.0526), the minimum panel size should be about 5 Mb based on the simulation results. Based on the simulated results, the panel sizes for the seven well-known commercially gene-targeted panels (< 1.5 Mb) did not conform to the high accuracy for the TMB-high and -low classifications. To test our hypothesis, we computed the accuracy of the TMB-high and -low classification for the patients with TMB between 9 and 11 mutations/Mb for the seven panels. The overall accuracies for the seven panels were between 0.4684 (OCAv3) and 0.5759 (OTML), indicating that about half of these patients were classified into the wrong group.

### TMB estimation using nonsynonymous mutations

**Figure 5:**
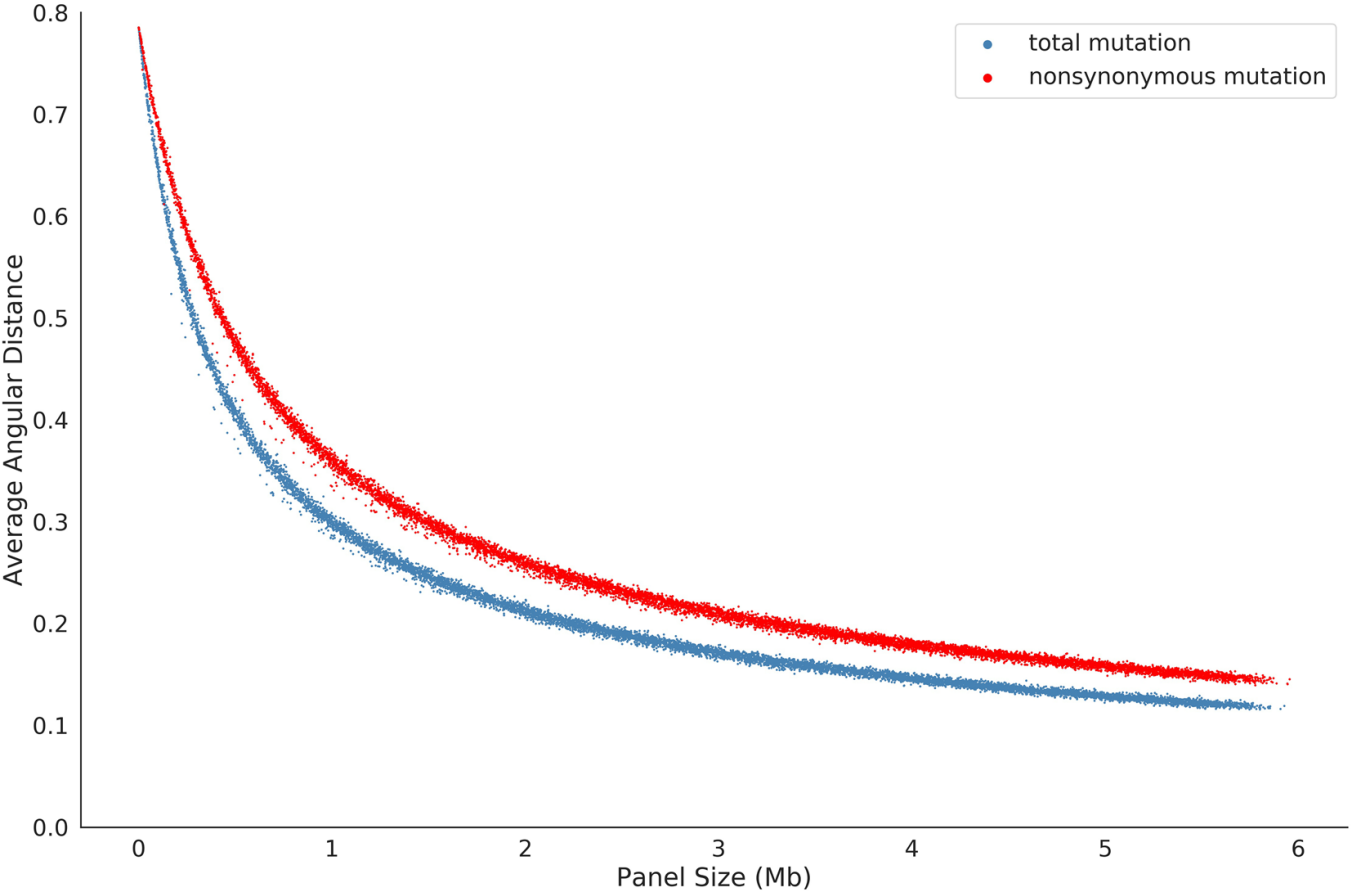
The average angular distance of the simulated sequencing panels in the hypermutation-included dataset using total point mutations (blue) and nonsynonymous mutations (red). The X axis is the size for each simulated panel, and the Y axis is the average angular distance estimated by the simulated panels. The estimation bias is higher when using nonsynonymous mutations as opposed to using total point mutations for the same panels.

The TMB defined by the F1CDx assay counts the number of total point mutations in coding regions of the selected genes. Nevertheless, some panels such as MSK-IMPACT and OTML only count the nonsynonymous mutations, as they believe that nonsynonymous mutations leading to the changes in amino acids will result in new neo-antigens that are considered to be foreign to the immune system^13,30^, and can be detected by the immune system. In addition, they argue that synonymous mutations should not be included in TMB estimation because the immune system has a higher likelihood of recognizing these alterations as normal^31^. To reconcile this problem, we compared the performance of the 10,000 simulated panels using total point mutations and nonsynonymous mutations, respectively (Fig. 5). Our in silico analysis indicates that the estimated bias is higher when using nonsynonymous mutations as opposed to using total point mutations. Therefore, a higher panel size is suggested when using nonsynonymous mutations to estimate the TMB of a patient.

## Discussion

With the development of gene-targeted panels enabling cost-effective estimation for more patients^32^, developing a statistical method to effectively evaluate and compare the performance of TMB estimation for different panels is necessary. Though the most popular method uses R-squared value by constructing a linear regression model to measure the correlation between panel-based TMB and WES-based TMB, our observations show that R-squared is inaccurate to evaluate the performance of the panels. To address the issue, we applied a new measurement, angular distance, for panel evaluation. By comprehensive comparison of the two measurements, we have demonstrated that angular distance is a more logical and efficient solution.

To quantify the correlation between the panel size and the estimated accuracy, we randomly simulated 10,000 panels with different sizes and analyzed their performance. Our simulation has demonstrated that panel size is an important factor that affects the accuracy of TMB estimation^28^. Higher estimated biases usually occur in lower-TMB patients. Therefore, for a patient with higher TMB, a smaller panel size sufficiently conforms to the WES-based TMB, whereas for a patient with lower TMB, a larger panel size is needed. In particular, based on the FDA approval of a 10 mutations/Mb threshold to select patients for ICB therapy, we computed the rate of bias in TMB estimation for patients with TMB between 9 and 11 mutations/Mb under different panel sizes. Our observation suggests that the panel sizes of the selected commercially gene-targeted panels do not conform to the need for accuracy for the TMB-high and -low classifications.

TMB is one of the most rapidly developing biomarkers for immunotherapy^10^, and studies have added to the evidence supporting the use of TMB to identify patients who are most likely to benefit from immunotherapy across a wide range of tumor types^33^. In particular, integrating TMB with other biomarkers has the potential to improve the predictive accuracy of TMB^34^. The growing number of gene-targeted panels being produced as well as the optimization of the statistical methods for panel evaluation help to harmonize the TMB quantification in clinical trials. For example, a recent study proposed that using “accuracy” to predict “high-TMB” status instead of R-squared to measure the performance of a panel^35^. Notably, these binary prediction methods are informative, but have their limitations. One issue is how to determine optimal TMB cutoffs for treatment. On one hand, different studies have assigned different cut-offs to delineate TMB-high and TMB-low status^14,15,22,29^; On the other hand, due to tumor heterogeneity, studies have illustrated that different cutoffs are needed for different cancer types^36^. Therefore, the classification may be controversial under different cutoffs, and may not accurately predict the outcome of a patient. In contrast, angular distance does not determine a patient’s TMB status. Instead, using angular distance and panel-based TMB, we can calculate the confidence interval of the WES-based TMB, and then predict the potential outcome for the patient based on the statistics of previous clinical trials, which will be more informative for clinical treatment decisions. In summary, our study provides a clear sketch for evaluating a gene-targeted panel, highlights the TMB’s utility as a biomarker of immunotherapy and helps to inform more personalized treatment plans for a patient.

## Methods

### TCGA mutation data

The WES somatic tumor mutation calling MAF file was downloaded from the Cancer Genome Atlas (TCGA)^37^. Mutation calls were made from Multi-Center Mutation Calling in Multiple Cancers (MC3) working group using seven software packages (MuTect, MuSE, VarScan2, Radia, Pindel, Somatic Sniper, Indelocator)^38^. In the MAF file, there were 10,223 patients across 33 different cancer types. The file containing protein-coding regions was downloaded from the Consensus Coding Sequence (CCDS) project^39^. The length of each gene was calculated from the column “cds_locations” in the CCDS file. According to the CCDS file, the total protein-coding region length is approximately 36.54 Mb, which was similar to the previous reports.

### TMB calculation

TMB is defined as the number of somatic mutations per megabase (Mb) of interrogated genomic sequence^36^. It can be calculated using the equation: TMB = The number of mutations in the target region/The total size of the target region (Mb). Since the protein-coding region provides the best association with cancer research and clinical benefit, whole exome sequencing (WES) is regarded as the “gold-standard” to measure TMB.

### Mutation type extraction

The MAF file contains 16 types of somatic mutations (Missense_Mutation, Silent, Nonsense_Mutation, Intron, 3'UTR, 5'UTR, Splice_Site, RNA, Frame_Shift_Ins, Frame_Shift_Del, In_Frame_Ins, Nonstop_Mutation, In_Frame_Del, 3'Flank, 5'Flank, Translation_Start_Site) flagged by variant calling software packages. Since TMB is defined as the mutations in the coding region in most studies, four types of mutations (Intron, RNA, 3'Flank, 5'Flank) outside the coding region are excluded. Considering the mutation types are different for the two FDA’s approval assays F1CDx (total point mutations in the coding region) and MSK-IMPACT (nonsynonymous mutations in the coding region), both of which are included in our study.

### Select gene-targeted panels

We totally selected seven well-known commercially gene-targeted panels to test the two measurements: R-squared and angular distance. Besides the FDA's approved genomic profiling tests F1CDx (324 genes, 1.2 Mb) and MKS-IMPACT (468 genes, 1.22 Mb), we also preferred Oncomine™ Comprehensive Assay v3M (OCAv3, 161 genes, 0.21 Mb exonic region and 0.14 Mb intronic region) , OncomineTM Tumor Mutation Load Assay (OTML, 409 genes, 1.2 Mb exonic region and 0.45 Mb intronic region)^40^, TruSight Oncology 500 (TSO500, 523 genes, 1.34 Mb exonic region and 0.61 Mb intronic region), TruSight Tumor 170 (TST170, 171 genes, 0.41 Mb exonic region and 0.12 Mb intronic region) and Human tumor mutational load assay (QIAseq, 486 genes, 1.26 Mb exonic region and 1.32 Mb intronic region).

### Generate gene-targeted panels in silico

In addition to selecting well-known commercially gene-targeted panels, we also randomly generated a total of 10,000 panels in silico. The file containing 19,600 genes with their coding regions was downloaded from the Consensus Coding Sequence (CCDS) project^39^. To limit the panel size, we set the maximum number of genes for each simulated panel to be 3,000. For each iteration, we first randomly generated a number *N* between 1 and 3,000 representing the number of genes in the corresponding panel. We then randomly selected *N* genes for the 19,600 genes. Therefore, the panel size for the simulated panel was the summation of the CDS region for these *N* genes. After the genes were confirmed, we counted the total number of somatic mutations in the coding region of these genes. The TMB of the simulated panel was obtained by dividing panel size into the total number of somatic mutations.

### Hypermutation definition

The TMB distribution of the downloaded TCGA dataset displays a long-tailed pattern. In majority of patients, their TMB is smaller than 10 mutations/Mb (Fig. 1A). In contrast, for patients of certain cancer types, such as malignant melanoma (exposure to ultraviolet light) and lung cancer (exposure to tobacco smoke), which are related to extensive exposure to carcinogens, their TMBs may exceed 400 mutations/Mb. To compare the different TMBs that affect the accuracy of estimation, we used a cutoff of 50 mutations/Mb^41^ (total point mutations in the coding region) to classify the hypermutated group and non-hypermutated group. In total, 308 patients were labeled hypermutated, and the remaining patients were labeled as non-hypermutated.

## Supplementary Information

Supplementary Information.

## Data Availability

The TCGA MC3 Public MAF file and the txt file are available at https://gdc.cancer.gov/about-data/publications/pancan-driver. The file containing protein-coding regions from the Consensus Coding Sequence (CCDS) project is available at https://ftp.ncbi.nlm.nih.gov/pub/CCDS/current_human.

## References

[1] Drake CG, Lipson EJ, Brahmer JR (2014). Breathing new life into immunotherapy: Review of melanoma, lung and kidney cancer. Nat. Rev. Clin. Oncol..

[2] Khalil DN, Smith EL, Brentjens RJ, Wolchok JD (2016). The future of cancer treatment: Immunomodulation, CARs and combination immunotherapy. Nat. Rev. Clin. Oncol..

[3] Leach DR, Krummel MF, Allison JP (1996). Enhancement of antitumor immunity by CTLA-4 blockade. Science.

[4] Tumeh PC (2014). PD-1 blockade induces responses by inhibiting adaptive immune resistance. Nature.

[5] Simoni Y (2018). Bystander CD8(+) T cells are abundant and phenotypically distinct in human tumour infiltrates. Nature.

[6] Lipson EJ (2013). Durable cancer regression off-treatment and effective reinduction therapy with an anti-PD-1 antibody. Clin. Cancer Res..

[7] Overman, M. J.* et al.* Nivolumab in patients with DNA mismatch repair deficient/microsatellite instability high metastatic colorectal cancer: Update from CheckMate 142. *J. Clin. Oncol.* **35**, 1182–1191 (2017). 10.1016/S1470-2045(17)30422-9 PMC6207072 28734759

[8] Alexandrov LB (2013). Signatures of mutational processes in human cancer. Nature.

[9] Yuan J (2016). Novel technologies and emerging biomarkers for personalized cancer immunotherapy. J. Immunother. Cancer.

[10] Chan TA (2019). Development of tumor mutation burden as an immunotherapy biomarker: Utility for the oncology clinic. Ann. Oncol..

[11] Kim, J. Y.* et al.* Tumor mutational burden and efficacy of immune checkpoint inhibitors: A systematic review and meta-analysis. *Cancers (Basel)* **11**, 1798 (2019). 10.3390/cancers11111798 PMC6895916 31731749

[12] Samstein RM (2019). Tumor mutational load predicts survival after immunotherapy across multiple cancer types. Nat. Genet..

[13] Snyder A (2014). Genetic basis for clinical response to CTLA-4 blockade in melanoma. N. Engl. J. Med..

[14] Rizvi NA (2015). Cancer immunology. Mutational landscape determines sensitivity to PD-1 blockade in non-small cell lung cancer. Science.

[15] Carbone DP (2017). First-line nivolumab in stage IV or recurrent non-small-cell lung cancer. N. Engl. J. Med..

[16] McGrail DJ (2021). High tumor mutation burden fails to predict immune checkpoint blockade response across all cancer types. Ann. Oncol..

[17] Mouliere F (2013). Circulating cell-free DNA from colorectal cancer patients may reveal high KRAS or BRAF mutation load. Transl. Oncol..

[18] Li X, Pasche B, Zhang W, Chen K (2018). Association of MUC16 mutation with tumor mutation load and outcomes in patients with gastric cancer. JAMA Oncol..

[19] McFarland CD, Korolev KS, Kryukov GV, Sunyaev SR, Mirny LA (2013). Impact of deleterious passenger mutations on cancer progression. Proc. Natl. Acad. Sci. USA.

[20] Zhang Y (2019). Genetic load and potential mutational meltdown in cancer cell populations. Mol. Biol. Evol..

[21] Johnson DB (2016). Targeted next generation sequencing identifies markers of response to PD-1 blockade. Cancer Immunol. Res..

[22] Chalmers ZR (2017). Analysis of 100,000 human cancer genomes reveals the landscape of tumor mutational burden. Genome Med..

[23] Rizvi H (2018). Molecular determinants of response to anti-programmed cell death (PD)-1 and anti-programmed death-ligand 1 (PD-L1) blockade in patients with non-small-cell lung cancer profiled with targeted next-generation sequencing. J. Clin. Oncol..

[24] Cheng DT (2015). Memorial Sloan Kettering-integrated mutation profiling of actionable cancer targets (MSK-IMPACT): A hybridization capture-based next-generation sequencing clinical assay for solid tumor molecular oncology. J. Mol. Diagn..

[25] Lawrence MS (2013). Mutational heterogeneity in cancer and the search for new cancer-associated genes. Nature.

[26] Melendez B, Van Campenhout C, Rorive S, Remmelink M, Salmon I, D'Haene N (2018). Methods of measurement for tumor mutational burden in tumor tissue. Transl. Lung Cancer Res..

[27] Wang Z (2019). Assessment of blood tumor mutational burden as a potential biomarker for immunotherapy in patients with non-small cell lung cancer with use of a next-generation sequencing cancer gene panel. JAMA Oncol..

[28] Buchhalter I (2019). Size matters: Dissecting key parameters for panel-based tumor mutational burden analysis. Int. J. Cancer.

[29] Hellmann MD (2018). Nivolumab plus ipilimumab in lung cancer with a high tumor mutational burden. N. Engl. J. Med..

[30] Rosenberg JE (2016). Atezolizumab in patients with locally advanced and metastatic urothelial carcinoma who have progressed following treatment with platinum-based chemotherapy: A single-arm, multicentre, phase 2 trial. Lancet.

[31] Stewart TJ, Abrams SI (2008). How tumours escape mass destruction. Oncogene.

[32] Fancello L, Gandini S, Pelicci PG, Mazzarella L (2019). Tumor mutational burden quantification from targeted gene panels: Major advancements and challenges. J. Immunother. Cancer.

[33] Allgauer M (2018). Implementing tumor mutational burden (TMB) analysis in routine diagnostics-a primer for molecular pathologists and clinicians. Transl. Lung Cancer Res..

[34] Cristescu, R.* et al.* Pan-tumor genomic biomarkers for PD-1 checkpoint blockade-based immunotherapy. *Science* **362**, eaar3593 (2018). 10.1126/science.aar3593 PMC6718162 30309915

[35] Wu HX, Wang ZX, Zhao Q, Wang F, Xu RH (2019). Designing gene panels for tumor mutational burden estimation: The need to shift from 'correlation' to 'accuracy'. J. Immunother. Cancer.

[36] Merino, D. M.* et al.* Establishing guidelines to harmonize tumor mutational burden (TMB): In silico assessment of variation in TMB quantification across diagnostic platforms: Phase I of the Friends of Cancer Research TMB Harmonization Project. *J. Immunother. Cancer* **8**, e000147 (2020). 10.1136/jitc-2019-000147 PMC7174078 32217756

[37] Cancer Genome Atlas Research N*, et al.* The Cancer Genome Atlas Pan-Cancer analysis project. *Nat. Genet.* **45**, 1113–1120 (2013). 10.1038/ng.2764 PMC3919969 24071849

[38] Ellrott K (2018). Scalable open science approach for mutation calling of tumor exomes using multiple genomic pipelines. Cell Syst..

[39] Pruitt KD (2009). The consensus coding sequence (CCDS) project: Identifying a common protein-coding gene set for the human and mouse genomes. Genome Res..

[40] Chaudhary R (2018). A scalable solution for tumor mutational burden from formalin-fixed, paraffin-embedded samples using the Oncomine Tumor Mutation Load Assay. Transl. Lung Cancer Res..

[41] Campbell BB (2017). Comprehensive analysis of hypermutation in human cancer. Cell.

