# Supplementary material for: Optimizing the evaluation of gene-targeted panels for tumor mutational burden estimation
Source: Sci Rep. 2021 Oct 26;11:21072. doi: 10.1038/s41598-021-00626-7 (PMC8548549; doi:10.1038/s41598-021-00626-7)
Supplement: Supplementary file 1 — Supplementary Information. [file 41598_2021_626_MOESM1_ESM.pdf]

Supplementary Information: optimizing the evaluation of gene-targeted panels for tumor mutational burden estimation

Yawei Li[1], Yuan Luo[1*]

[1] Department of Preventive Medicine, Northwestern University, Feinberg School of Medicine, Chicago, IL 60611, USA

* Corresponding author:

Email: yuan.luo@northwestern.edu

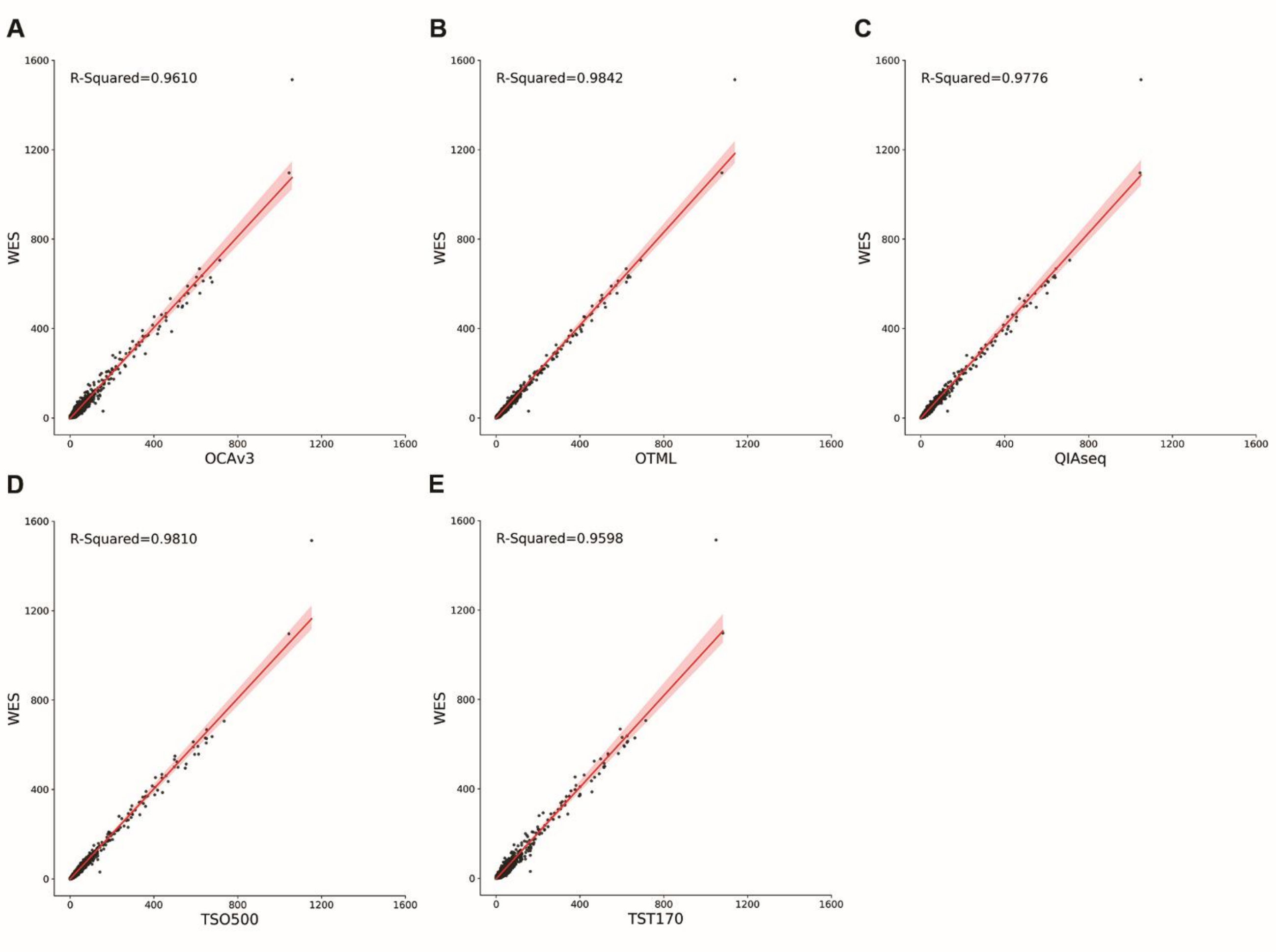

Figure S1. Linear fit with 95% confidence intervals of panel-based TMB estimated by sequencing panels OCAv3 (A), OTML (B), QIAseq (C), TSO500 (D) and TST170 (E) against WES-based TMB in the hypermutation-included dataset.

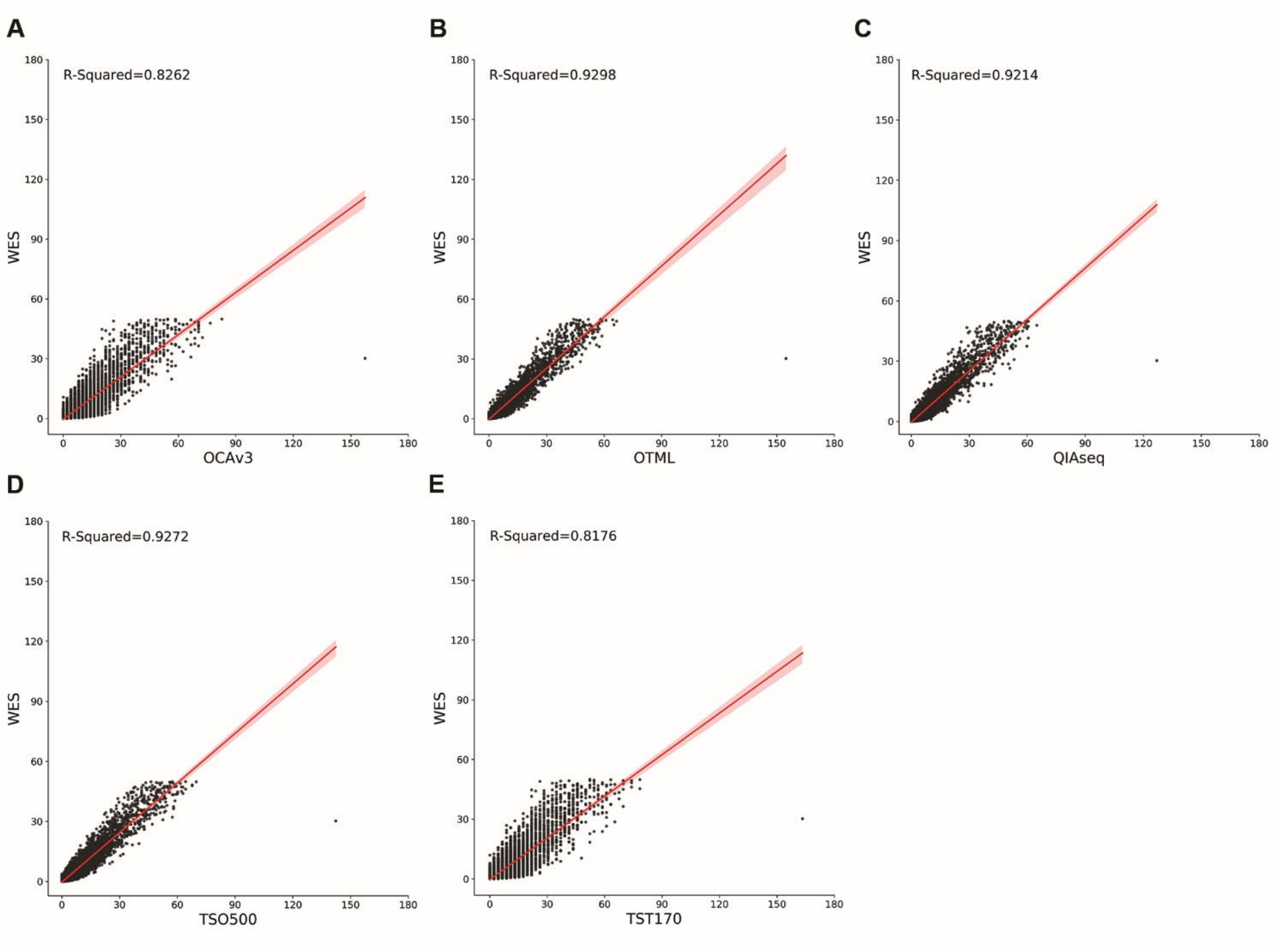

Figure S2. Linear fit with 95% confidence intervals of panel-based TMB estimated by sequencing panels OCAv3 (A), OTML (B), QIAseq (C), TSO500 (D) and TST170 (E) against WES-based TMB in the non-hypermutation dataset.

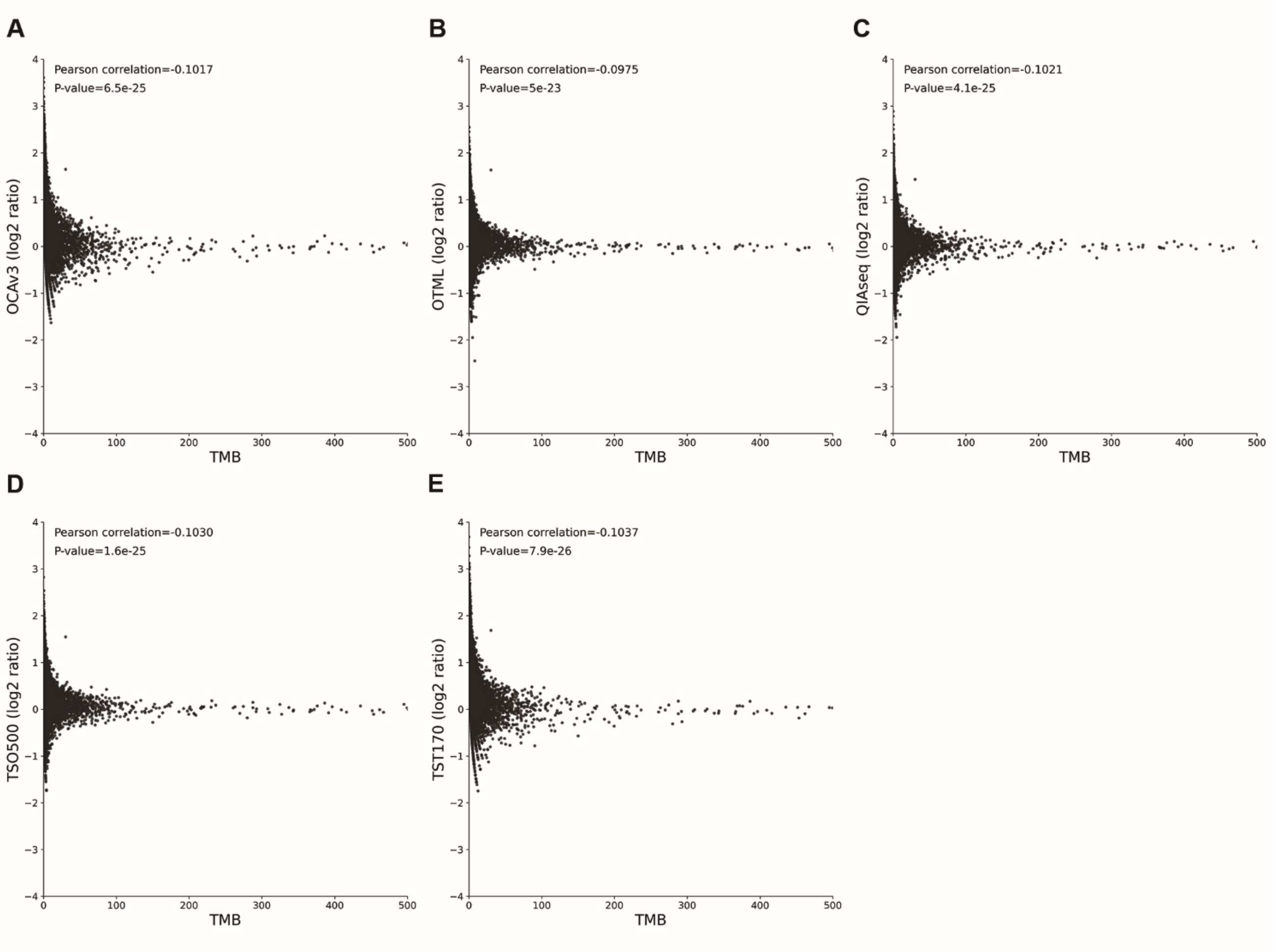

Figure S3. The correlation between the TMB size and the estimated bias for sequencing panels OCAv3 (Pearson correlation: -0.1017, P-value: 6.5×10^{-25}) (A), OTML (Pearson correlation: -0.0975, P-value: 5.0×10^{-23}) (B), QIAseq (Pearson correlation: -0.1021, P-value: 4.1×10^{-25}) (C), TSO500 (Pearson correlation: -0.1030, P-value: 1.6×10^{-25}) (D) and TST170 (Pearson correlation: -0.1037, P-value: 7.9×10^{-26}) (E). The X axis is the WES-based TMB of the patients and the Y axis is calculated as the panel-based TMB divided by the WES-based TMB for each patient. A log-2 scale is used for the Y axis of the graph. Patients with TMB >500 are not shown.

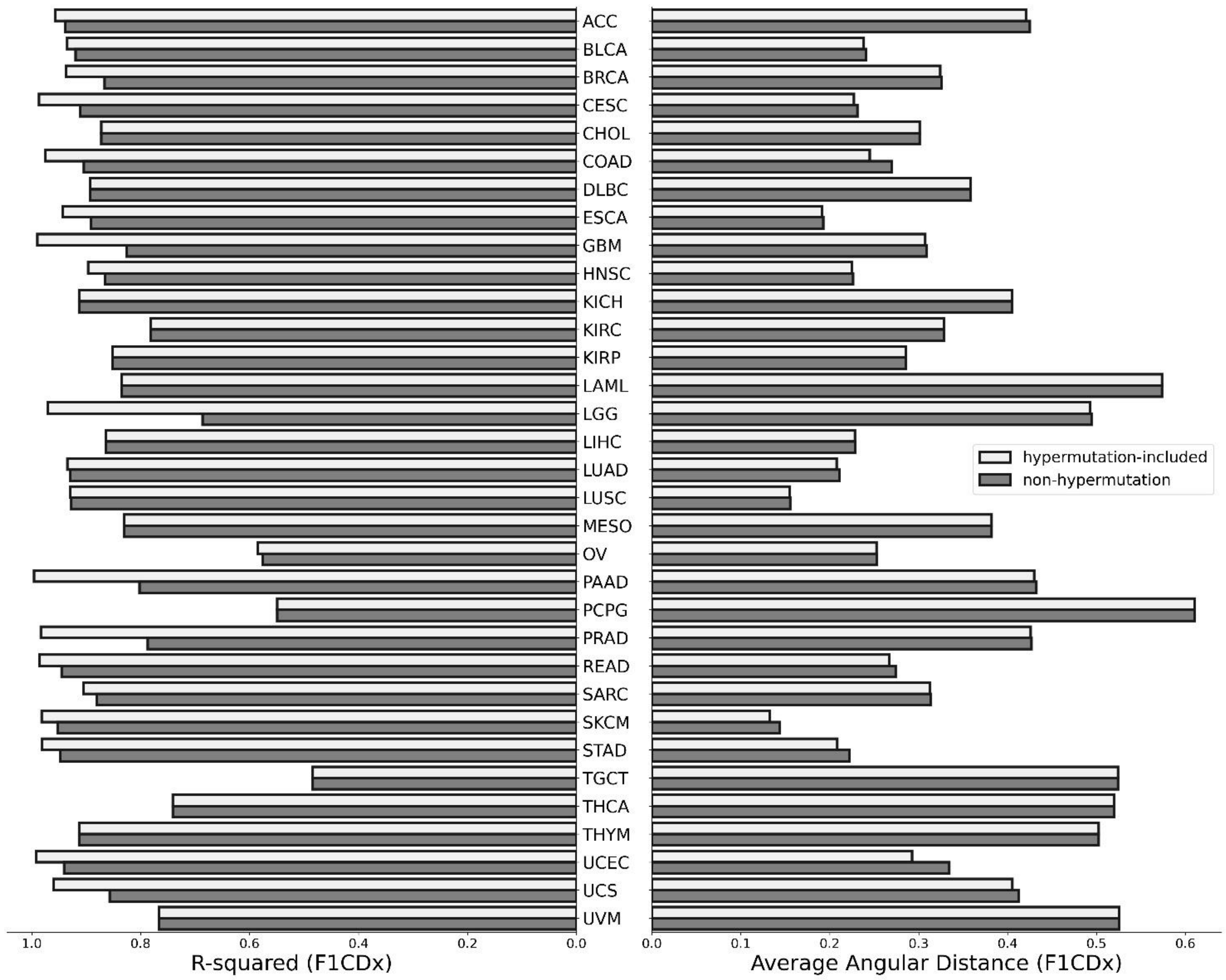

Figure S4. Bar plot of R-squared (left) and average angular distances (right) between hypermutation-included dataset (white) and non-hypermutation dataset (grey) achieved by the panel F1CDx across the 33 cancer types.

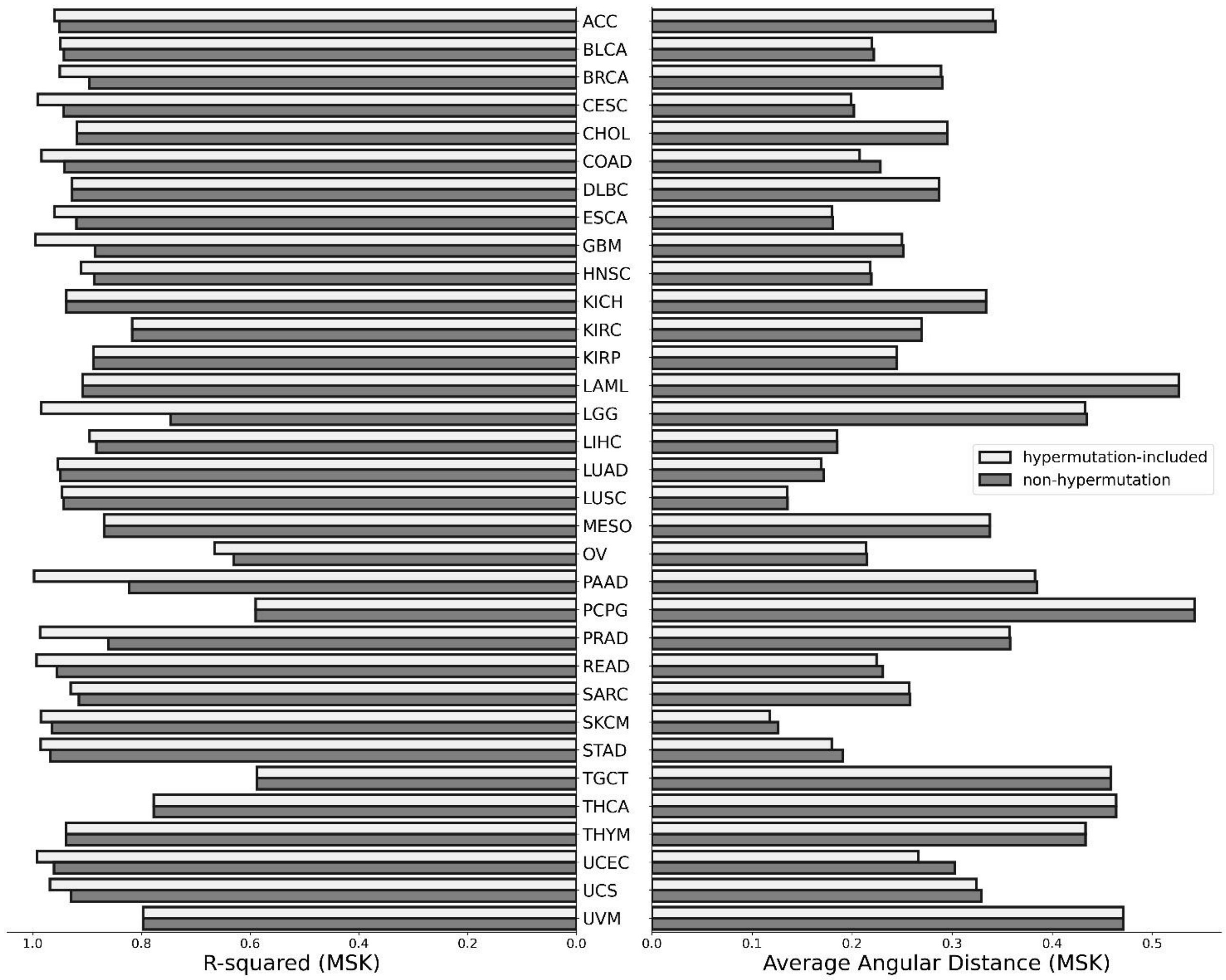

Figure S5. Bar plot of R-squared (left) and average angular distances (right) between hypermutation-included dataset (white) and non-hypermutation dataset (grey) achieved by the panel MSK-IMPACT across the 33 cancer types.

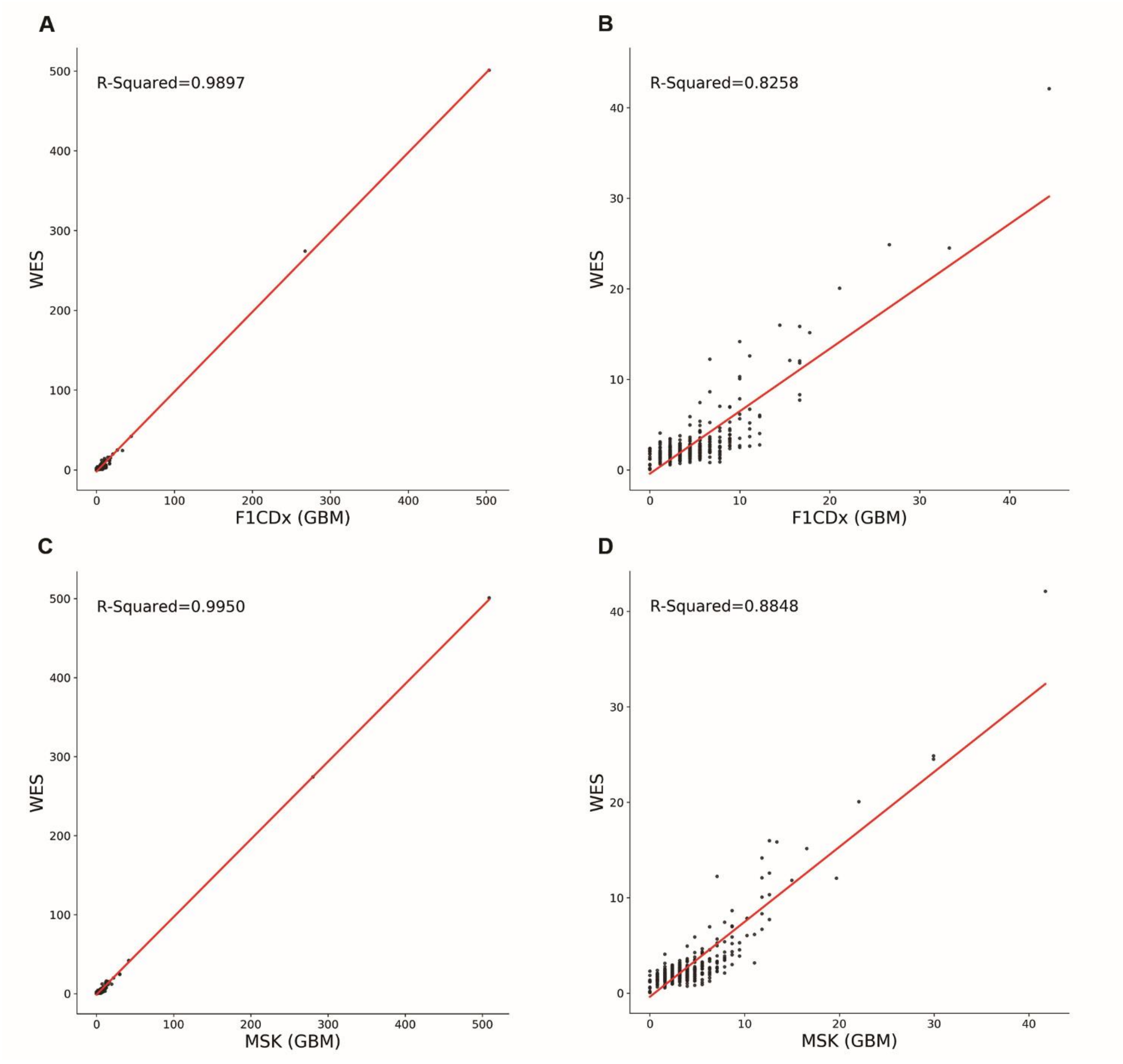

Figure S6. Linear fit of panel-based TMB estimated by F1CDx (A and B) and MSK (C and D) against WES-based TMB of cancer type GBM in the hypermutation-included dataset (A and C) and the non-hypermutation dataset (B and D).

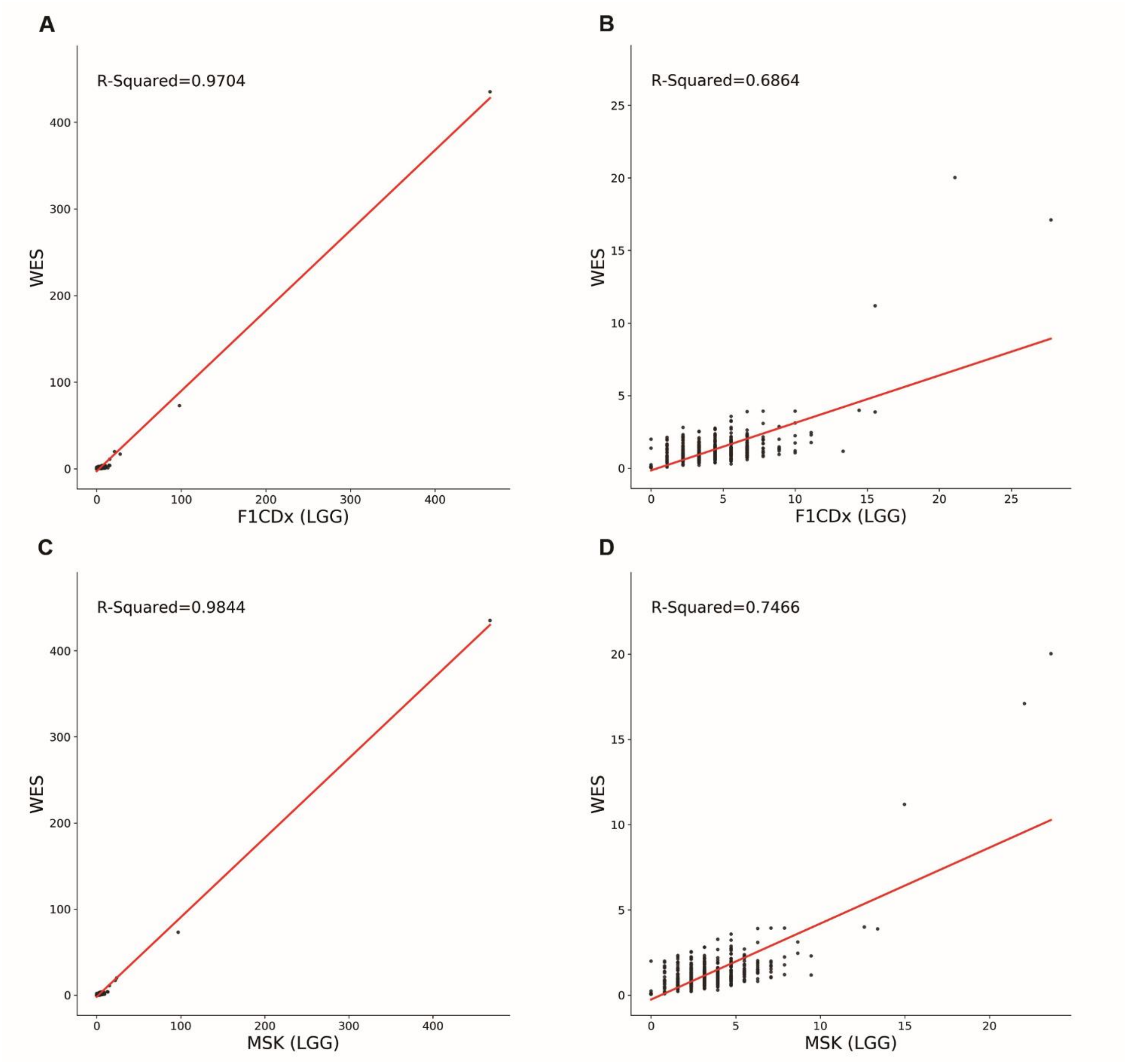

Figure S7. Linear fit of panel-based TMB estimated by F1CDx (A and B) and MSK (C and D) against WES-based TMB of cancer type LGG in the hypermutation-included dataset (A and C) and the non-hypermutation dataset (B and D).

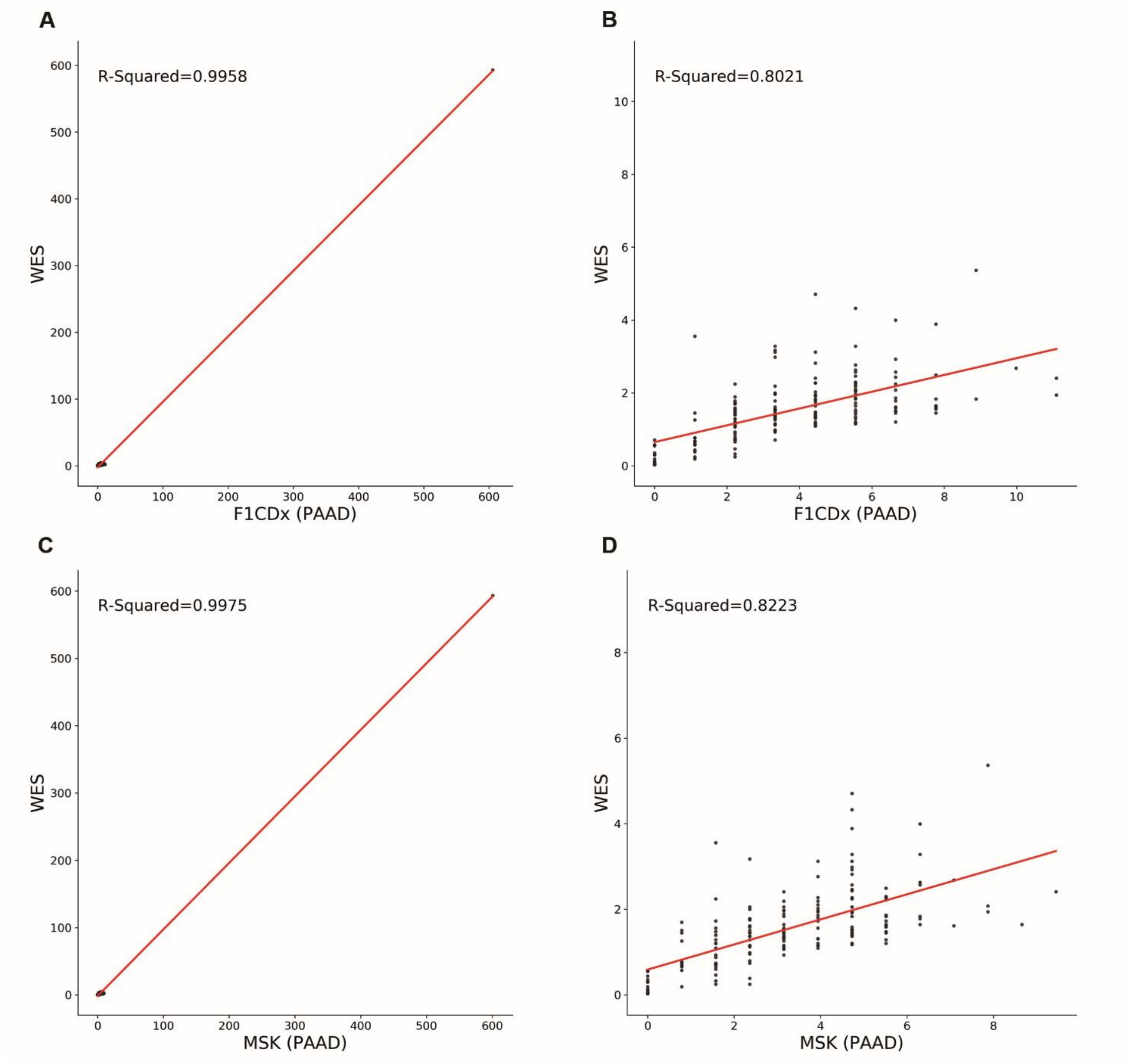

Figure S8. Linear fit of panel-based TMB estimated by F1CDx (A and B) and MSK (C and D) against WES-based TMB of cancer type PAAD in the hypermutation-included dataset (A and C) and the non-hypermutation dataset (B and D).

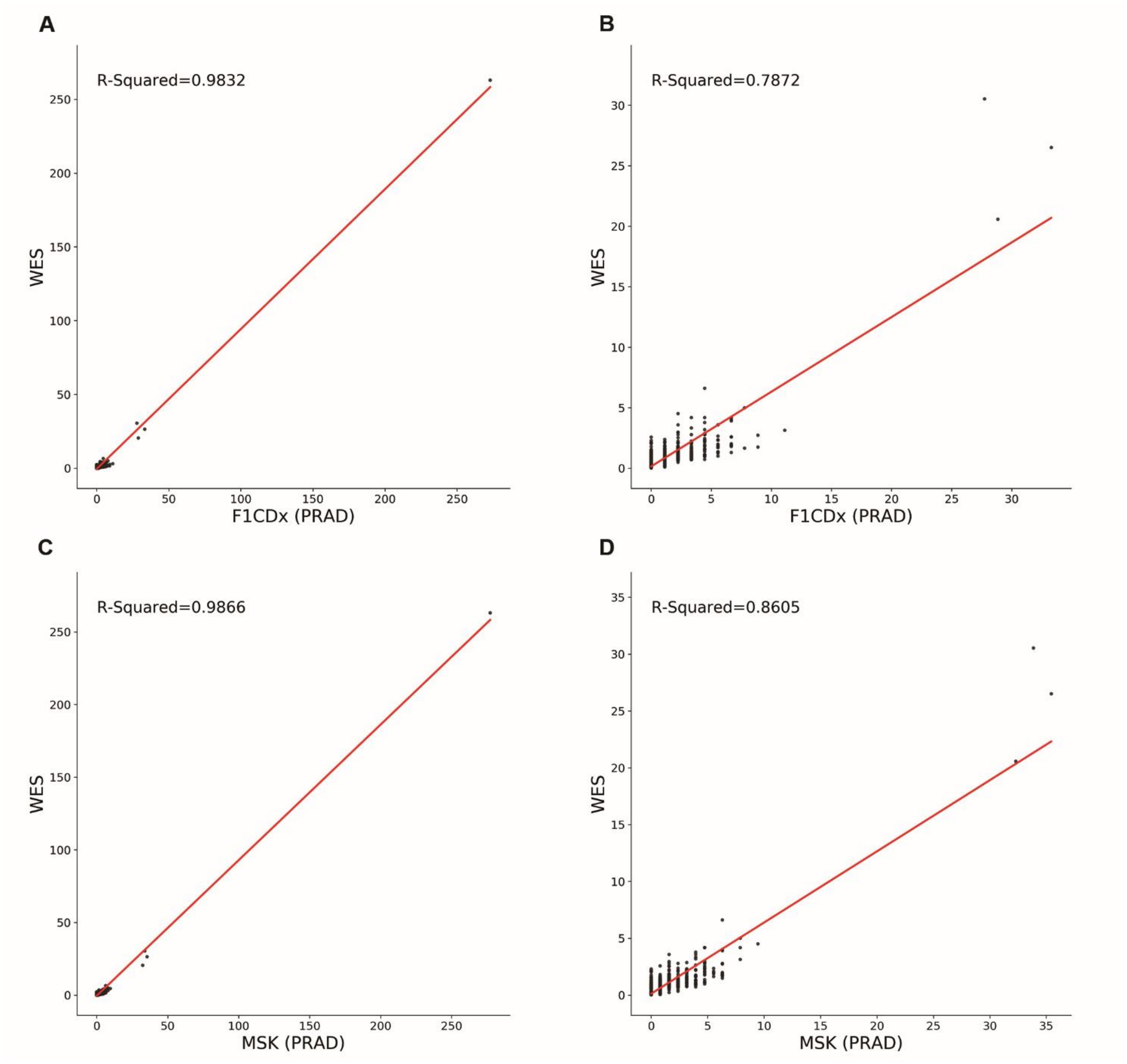

Figure S9. Linear fit of panel-based TMB estimated by F1CDx (A and B) and MSK (C and D) against WES-based TMB of cancer type PRAD in the hypermutation-included dataset (A and C) and the non-hypermutation dataset (B and D).

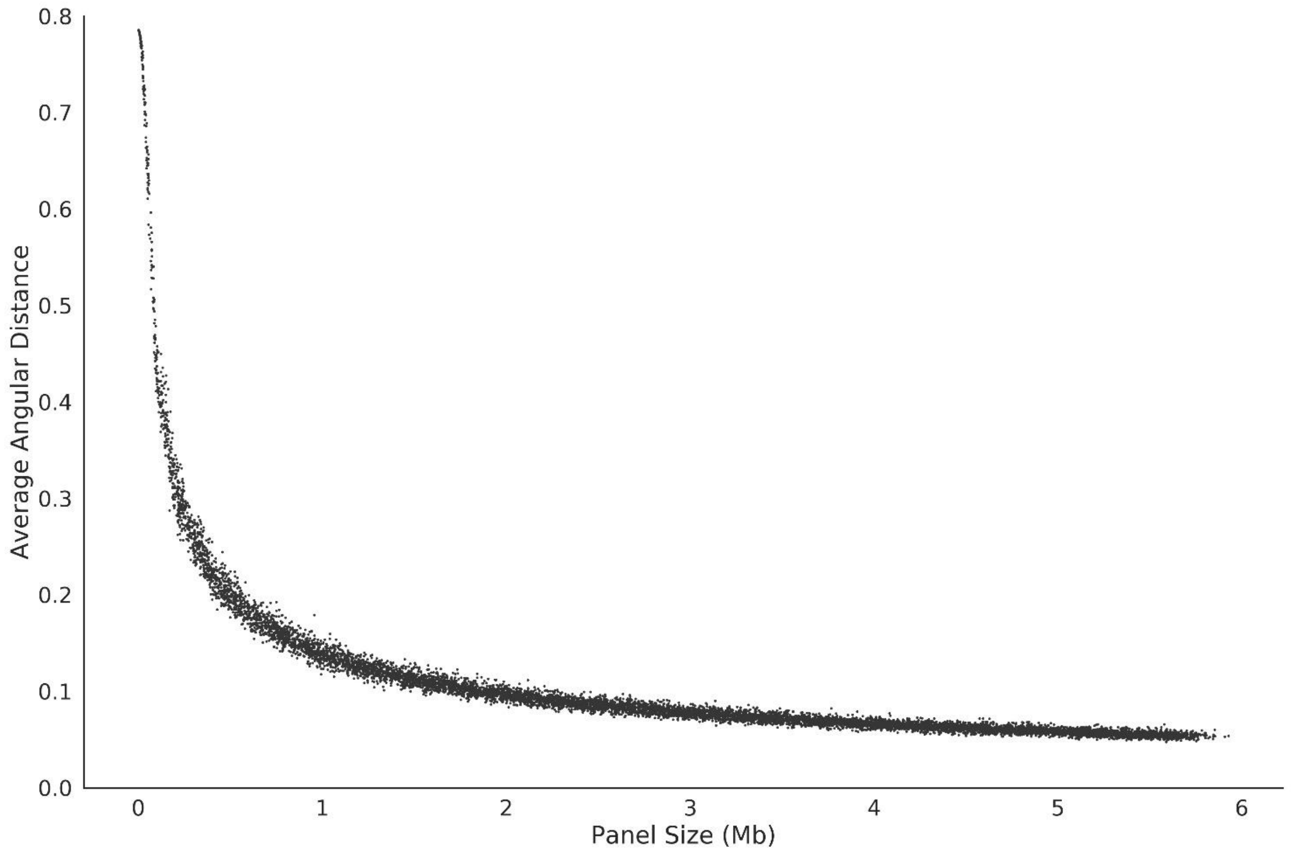

Figure S10. The average angular distance of the simulated sequencing panels of patients with TMBs between 9 and 11.

The X axis is the size of each simulated panel, and the Y axis is the average angular distance.

Table S1. The R-squared values for the selected panels across the 33 cancer types in the hypermutation-included dataset.

	Patients	F1CDx	MSK	OTML	OCAv3	TST170	TSO500	QIAseq	Average
ACC	92	0.9572	0.9599	0.9798	0.9093	0.9387	0.9805	0.9747	0.9571
BLCA	411	0.9350	0.9495	0.9656	0.8963	0.9003	0.9541	0.9500	0.9358
BRCA	1020	0.9368	0.9504	0.9703	0.8911	0.8846	0.9540	0.9539	0.9344
CESC	289	0.9866	0.9909	0.9908	0.9742	0.9700	0.9923	0.9884	0.9847
CHOL	36	0.8724	0.9183	0.9088	0.8071	0.7823	0.9109	0.9515	0.8788
COAD	406	0.9753	0.9840	0.9902	0.9700	0.9576	0.9859	0.9857	0.9784
DLBC	37	0.8929	0.9274	0.9416	0.8026	0.7534	0.9299	0.9049	0.8790
ESCA	184	0.9431	0.9606	0.9719	0.8708	0.9134	0.9662	0.9581	0.9406
GBM	393	0.9897	0.9950	0.9903	0.9783	0.9739	0.9943	0.9947	0.9880
HNSC	507	0.8966	0.9109	0.9403	0.8200	0.8108	0.9222	0.9256	0.8895
KICH	66	0.9132	0.9381	0.9565	0.8615	0.8204	0.9279	0.9161	0.9048
KIRC	369	0.7818	0.8173	0.8396	0.6584	0.6607	0.8570	0.8824	0.7853
KIRP	281	0.8522	0.8886	0.8748	0.6959	0.7109	0.9080	0.8776	0.8297
LAML	140	0.8351	0.9080	0.9289	0.8376	0.7720	0.9299	0.9240	0.8765
LGG	512	0.9704	0.9844	0.9888	0.9281	0.9443	0.9874	0.9839	0.9696
LIHC	363	0.8638	0.8956	0.9223	0.7777	0.7647	0.9122	0.8888	0.8607
LUAD	567	0.9345	0.9541	0.9666	0.8918	0.8941	0.9677	0.9654	0.9392
LUSC	485	0.9295	0.9463	0.9558	0.8891	0.8829	0.9549	0.9508	0.9299
MESO	82	0.8305	0.8681	0.8151	0.6755	0.7034	0.8683	0.8023	0.7947
OV	412	0.5854	0.6653	0.6072	0.6371	0.6418	0.6862	0.7317	0.6507
PAAD	177	0.9958	0.9975	0.9981	0.9870	0.9866	0.9981	0.9977	0.9944
PCPG	179	0.5495	0.5900	0.6083	0.4826	0.2842	0.6084	0.5900	0.5304
PRAD	497	0.9832	0.9866	0.9903	0.9515	0.9635	0.9884	0.9877	0.9787
READ	150	0.9856	0.9929	0.9945	0.9863	0.9823	0.9940	0.9951	0.9901
SARC	236	0.9047	0.9302	0.9448	0.8161	0.8331	0.9443	0.9400	0.9019
SKCM	466	0.9818	0.9848	0.9851	0.9717	0.9658	0.9856	0.9788	0.9791
STAD	439	0.9814	0.9852	0.9905	0.9627	0.9651	0.9879	0.9873	0.9800
TGCT	145	0.4840	0.5877	0.5824	0.3821	0.4117	0.6293	0.6037	0.5258
THCA	492	0.7412	0.7774	0.7212	0.5235	0.5114	0.7921	0.8044	0.6959
THYM	123	0.9133	0.9389	0.9477	0.8390	0.9118	0.9463	0.9219	0.9170
UCEC	530	0.9919	0.9919	0.9972	0.9888	0.9901	0.9942	0.9956	0.9928
UCS	57	0.9598	0.9683	0.9851	0.9080	0.9392	0.9762	0.9715	0.9583
UVM	80	0.7667	0.7973	0.7967	0.5794	0.5793	0.8080	0.8791	0.7438

Table S2. The R-squared values for the selected panels across the 33 cancer types in the non-hypermutation dataset.

	Patients	F1CDx	MSK	OTML	OCAv3	TST170	TSO500	QIAseq	Average
ACC	91	0.9383	0.9512	0.9463	0.8892	0.9098	0.9621	0.9563	0.9362
BLCA	405	0.9196	0.9425	0.9544	0.8664	0.8695	0.9475	0.9460	0.9209
BRCA	1014	0.8664	0.8957	0.9217	0.8210	0.7927	0.9061	0.9041	0.8725
CESC	282	0.9113	0.9434	0.9523	0.8443	0.8420	0.9468	0.9426	0.9118
CHOL	36	0.8724	0.9183	0.9088	0.8071	0.7823	0.9109	0.9515	0.8788
COAD	360	0.9045	0.9417	0.9453	0.8812	0.8301	0.9522	0.9399	0.9136
DLBC	37	0.8929	0.9274	0.9416	0.8026	0.7534	0.9299	0.9049	0.8790
ESCA	182	0.8913	0.9196	0.9468	0.8429	0.8473	0.9291	0.9291	0.9009
GBM	391	0.8258	0.8848	0.8745	0.6666	0.6760	0.8988	0.8881	0.8164
HNSC	503	0.8657	0.8869	0.9310	0.8004	0.7807	0.9052	0.9070	0.8681
KICH	66	0.9132	0.9381	0.9565	0.8615	0.8204	0.9279	0.9161	0.9048
KIRC	369	0.7818	0.8173	0.8396	0.6584	0.6607	0.8570	0.8824	0.7853
KIRP	281	0.8522	0.8886	0.8748	0.6959	0.7109	0.9080	0.8776	0.8297
LAML	140	0.8351	0.9080	0.9289	0.8376	0.7720	0.9299	0.9240	0.8765
LGG	510	0.6864	0.7466	0.7321	0.5705	0.6191	0.7591	0.7456	0.6942
LIHC	362	0.8636	0.8829	0.9123	0.7484	0.7302	0.9001	0.8886	0.8466
LUAD	553	0.9292	0.9496	0.9613	0.8917	0.8980	0.9601	0.9568	0.9352
LUSC	482	0.9275	0.9433	0.9548	0.8717	0.8690	0.9539	0.9464	0.9238
MESO	82	0.8305	0.8681	0.8151	0.6755	0.7034	0.8683	0.8023	0.7947
OV	411	0.5760	0.6301	0.5871	0.6544	0.6464	0.6556	0.6917	0.6345
PAAD	176	0.8021	0.8223	0.8443	0.7622	0.7518	0.8474	0.8373	0.8096
PCPG	179	0.5495	0.5900	0.6083	0.4826	0.2842	0.6084	0.5900	0.5304
PRAD	496	0.7872	0.8605	0.8359	0.6802	0.6105	0.8792	0.8483	0.7860
READ	145	0.9450	0.9559	0.9509	0.9059	0.8627	0.9531	0.9552	0.9327
SARC	235	0.8801	0.9150	0.9273	0.7632	0.7840	0.9312	0.9250	0.8751
SKCM	380	0.9529	0.9646	0.9728	0.9173	0.9201	0.9741	0.9650	0.9524
STAD	399	0.9477	0.9675	0.9618	0.9131	0.8966	0.9685	0.9693	0.9464
TGCT	145	0.4840	0.5877	0.5824	0.3821	0.4117	0.6293	0.6037	0.5258
THCA	492	0.7412	0.7774	0.7212	0.5235	0.5114	0.7921	0.8044	0.6959
THYM	123	0.9133	0.9389	0.9477	0.8390	0.9118	0.9463	0.9219	0.9170
UCEC	452	0.9405	0.9608	0.9661	0.8953	0.8774	0.9662	0.9574	0.9377
UCS	56	0.8570	0.9295	0.9220	0.7606	0.7691	0.9152	0.9416	0.8707
UVM	80	0.7667	0.7973	0.7967	0.5794	0.5793	0.8080	0.8791	0.7438

Table S3. The TMB distribution of the patients across the 33 cancer types in the hypermutation-included dataset and the non-hypermutation dataset.

	Hypermutation-included dataset				Non-hypermutation dataset			
	Patients	Average TMB	Variance	Average R-squared	Patients	Average TMB	Variance	Average R-squared
ACC	92	3.5641	92.7679	0.9572	91	2.7383	30.3645	0.9362
BLCA	411	10.3368	146.2964	0.9358	405	9.3021	61.7174	0.9209
BRCA	1020	3.6231	115.7124	0.9344	1014	2.9565	20.7090	0.8725
CESC	289	9.8559	1190.8537	0.9847	282	6.5747	55.1981	0.9118
CHOL	36	3.4210	31.4150	0.8788	36	3.4210	31.4150	0.8788
COAD	406	18.6974	1598.2371	0.9784	360	7.5793	101.7236	0.9136
DLBC	37	5.7577	11.2690	0.8790	37	5.7577	11.2690	0.8790
ESCA	184	7.1931	83.6741	0.9406	182	6.3769	22.0772	0.9009
GBM	393	4.8785	828.9696	0.9880	391	2.9202	11.8947	0.8164
HNSC	507	6.7888	81.0797	0.8895	503	6.1664	29.9485	0.8681
KICH	66	1.3784	12.2658	0.9048	66	1.3784	12.2658	0.9048
KIRC	369	2.3735	5.3425	0.7853	369	2.3735	5.3425	0.7853
KIRP	281	3.4524	14.5080	0.8297	281	3.4524	14.5080	0.8297
LAML	140	1.6251	22.5120	0.8765	140	1.6251	22.5120	0.8765
LGG	512	2.2847	379.5072	0.9696	510	1.2968	1.7796	0.6942
LIHC	363	4.6038	21.6279	0.8607	362	4.4599	14.1470	0.8466
LUAD	567	11.8590	156.6165	0.9392	553	10.6279	96.7970	0.9352
LUSC	485	11.8147	97.5379	0.9299	482	11.3477	59.5527	0.9238
MESO	82	1.3284	1.5565	0.7947	82	1.3284	1.5565	0.7947
OV	412	3.5539	22.3054	0.6507	411	3.3677	8.0418	0.6345
PAAD	177	4.8755	1978.2643	0.9944	176	1.5331	0.8100	0.8096
PCPG	179	0.4234	0.0657	0.5304	179	0.4234	0.0657	0.5304
PRAD	497	1.8964	142.0991	0.9787	496	1.3698	4.3149	0.7860
READ	150	12.4662	2621.0157	0.9901	145	4.7168	28.3302	0.9327
SARC	236	3.6944	69.2797	0.9019	235	3.2979	32.3279	0.8751
SKCM	466	33.6465	5908.8625	0.9791	380	17.5447	161.9328	0.9524
STAD	439	15.2959	997.6067	0.9800	399	8.1392	109.7675	0.9464
TGCT	145	0.6851	0.1390	0.5258	145	0.6851	0.1390	0.5258
THCA	492	0.6778	1.8259	0.6959	492	0.6778	1.8259	0.6959
THYM	123	1.0945	7.1669	0.9170	123	1.0945	7.1669	0.9170
UCEC	530	48.8032	16212.2170	0.9928	452	9.0860	139.9797	0.9377
UCS	57	4.9978	349.2783	0.9583	56	2.5711	13.8692	0.8707
UVM	80	0.6620	3.1608	0.7438	80	0.6620	3.1608	0.7438

Table S4. The average angular distances for the selected panels across the 33 cancer types in the hypermutation-included dataset.

	Patients	F1CDx	MSK	OTML	OCAv3	TST170	TSO500	QIAseq	Average
ACC	92	0.4206	0.3405	0.3200	0.4933	0.5218	0.3373	0.3653	0.3998
BLCA	411	0.2381	0.2196	0.1758	0.2753	0.2816	0.2042	0.2053	0.2286
BRCA	1020	0.3241	0.2887	0.2586	0.4010	0.3987	0.2769	0.2776	0.3179
CESC	289	0.2270	0.1988	0.1708	0.2994	0.3007	0.1822	0.1903	0.2242
CHOL	36	0.3009	0.2952	0.2754	0.3641	0.3859	0.2733	0.2677	0.3089
COAD	406	0.2448	0.2074	0.1882	0.2610	0.3384	0.1943	0.1938	0.2326
DLBC	37	0.3581	0.2869	0.2141	0.2412	0.2382	0.2938	0.2540	0.2695
ESCA	184	0.1915	0.1797	0.1688	0.2551	0.2448	0.1807	0.1630	0.1977
GBM	393	0.3072	0.2498	0.2456	0.3928	0.3967	0.2375	0.2577	0.2982
HNSC	507	0.2249	0.2179	0.1919	0.2992	0.2994	0.2086	0.2046	0.2352
KICH	66	0.4049	0.3338	0.3279	0.5633	0.5715	0.3207	0.3062	0.4040
KIRC	369	0.3285	0.2691	0.2445	0.3960	0.3990	0.2620	0.2627	0.3088
KIRP	281	0.2853	0.2446	0.2221	0.3855	0.3918	0.2347	0.2432	0.2867
LAML	140	0.5734	0.5261	0.5091	0.6289	0.6495	0.5079	0.5300	0.5607
LGG	512	0.4927	0.4329	0.4010	0.5527	0.5379	0.4148	0.4297	0.4660
LIHC	363	0.2281	0.1847	0.1690	0.2945	0.3009	0.1815	0.1910	0.2214
LUAD	567	0.2077	0.1688	0.1555	0.2509	0.2470	0.1615	0.1624	0.1934
LUSC	485	0.1550	0.1350	0.1336	0.1976	0.1938	0.1317	0.1326	0.1542
MESO	82	0.3818	0.3376	0.3224	0.4719	0.5141	0.3110	0.3176	0.3795
OV	412	0.2524	0.2140	0.2106	0.3097	0.3082	0.2112	0.2105	0.2452
PAAD	177	0.4298	0.3825	0.3410	0.5472	0.5467	0.3574	0.3636	0.4240
PCPG	179	0.6100	0.5419	0.5193	0.6963	0.7146	0.5339	0.5265	0.5918
PRAD	497	0.4258	0.3571	0.3293	0.5561	0.5853	0.3363	0.3558	0.4208
READ	150	0.2667	0.2242	0.2451	0.3039	0.4079	0.2093	0.2173	0.2678
SARC	236	0.3125	0.2569	0.2193	0.4174	0.4082	0.2389	0.2389	0.2989
SKCM	466	0.1326	0.1175	0.1085	0.1708	0.1694	0.1025	0.1186	0.1314
STAD	439	0.2082	0.1795	0.1812	0.2674	0.2698	0.1862	0.1752	0.2097
TGCT	145	0.5240	0.4585	0.4215	0.6562	0.6594	0.4116	0.4431	0.5106
THCA	492	0.5197	0.4637	0.4368	0.6204	0.6316	0.4371	0.4403	0.5071
THYM	123	0.5024	0.4331	0.4180	0.6406	0.6251	0.4064	0.4422	0.4954
UCEC	530	0.2924	0.2661	0.2148	0.3574	0.3473	0.2578	0.2561	0.2845
UCS	57	0.4053	0.3240	0.2630	0.4870	0.4671	0.3284	0.3259	0.3715
UVM	80	0.5251	0.4706	0.4244	0.6226	0.6193	0.4475	0.4353	0.5064

Table S5. The average angular distances for the selected panels across the 33 cancer types in the non-hypermutation dataset.

	Patients	F1CDx	MSK	OTML	OCAv3	TST170	TSO500	QIAseq	Average
ACC	91	0.4248	0.3431	0.3234	0.4977	0.5269	0.3410	0.3689	0.4037
BLCA	405	0.2406	0.2216	0.1777	0.2776	0.2839	0.2060	0.2073	0.2307
BRCA	1014	0.3257	0.2901	0.2600	0.4028	0.4006	0.2782	0.2789	0.3195
CESC	282	0.2310	0.2016	0.1736	0.3052	0.3066	0.1852	0.1939	0.2281
CHOL	36	0.3009	0.2952	0.2754	0.3641	0.3859	0.2733	0.2677	0.3089
COAD	360	0.2695	0.2281	0.2081	0.2861	0.3727	0.2131	0.2127	0.2557
DLBC	37	0.3581	0.2869	0.2141	0.2412	0.2382	0.2938	0.2540	0.2695
ESCA	182	0.1927	0.1807	0.1698	0.2571	0.2465	0.1815	0.1645	0.1989
GBM	391	0.3088	0.2510	0.2467	0.3946	0.3986	0.2386	0.2589	0.2996
HNSC	503	0.2262	0.2191	0.1933	0.3007	0.3013	0.2097	0.2057	0.2366
KICH	66	0.4049	0.3338	0.3279	0.5633	0.5715	0.3207	0.3062	0.4040
KIRC	369	0.3285	0.2691	0.2445	0.3960	0.3990	0.2620	0.2627	0.3088
KIRP	281	0.2853	0.2446	0.2221	0.3855	0.3918	0.2347	0.2432	0.2867
LAML	140	0.5734	0.5261	0.5091	0.6289	0.6495	0.5079	0.5300	0.5607
LGG	510	0.4943	0.4343	0.4023	0.5545	0.5396	0.4161	0.4311	0.4674
LIHC	362	0.2285	0.1851	0.1693	0.2952	0.3017	0.1819	0.1912	0.2218
LUAD	553	0.2108	0.1713	0.1581	0.2543	0.2499	0.1645	0.1654	0.1963
LUSC	482	0.1553	0.1354	0.1341	0.1986	0.1946	0.1321	0.1330	0.1547
MESO	82	0.3818	0.3376	0.3224	0.4719	0.5141	0.3110	0.3176	0.3795
OV	411	0.2530	0.2145	0.2110	0.3104	0.3089	0.2117	0.2109	0.2458
PAAD	176	0.4322	0.3846	0.3429	0.5503	0.5498	0.3594	0.3657	0.4264
PCPG	179	0.6100	0.5419	0.5193	0.6963	0.7146	0.5339	0.5265	0.5918
PRAD	496	0.4266	0.3577	0.3299	0.5571	0.5865	0.3370	0.3565	0.4216
READ	145	0.2739	0.2306	0.2525	0.3125	0.4200	0.2153	0.2236	0.2755
SARC	235	0.3133	0.2575	0.2197	0.4186	0.4094	0.2395	0.2394	0.2996
SKCM	380	0.1436	0.1259	0.1193	0.1844	0.1829	0.1127	0.1280	0.1424
STAD	399	0.2220	0.1907	0.1946	0.2852	0.2876	0.1981	0.1868	0.2236
TGCT	145	0.5240	0.4585	0.4215	0.6562	0.6594	0.4116	0.4431	0.5106
THCA	492	0.5197	0.4637	0.4368	0.6204	0.6316	0.4371	0.4403	0.5071
THYM	123	0.5024	0.4331	0.4180	0.6406	0.6251	0.4064	0.4422	0.4954
UCEC	452	0.3343	0.3026	0.2462	0.4087	0.3976	0.2937	0.2938	0.3253
UCS	56	0.4122	0.3292	0.2676	0.4953	0.4740	0.3342	0.3314	0.3777
UVM	80	0.5251	0.4706	0.4244	0.6226	0.6193	0.4475	0.4353	0.5064
